# Interfacing Broad-Spectrum Semiconductors with Hydrogenases for Semi-Artificial Solar Reforming of Cellulose

**DOI:** 10.1021/jacs.6c03439

**Published:** 2026-04-28

**Authors:** Ming Shi, Yongpeng Liu, Ariffin Bin Mohamad Annuar, Sophie Webb, Ross D. Milton, Rengui Li, Can Li, Erwin Reisner

**Affiliations:** † Yusuf Hamied Department of Chemistry, 2152 University of Cambridge, Cambridge, CB2 1EW, U.K.; ‡ State Key Laboratory of Catalysis, Dalian National Laboratory for Clean Energy, Dalian Institute of Chemical Physics, Chinese Academy of Sciences, Dalian 116023, China; § Department of Inorganic and Analytical Chemistry, University of Geneva, 1211, Geneva 4, Switzerland; ∥ National Centre of Competence in Research (NCCR) Catalysis, University of Geneva, 1211, Geneva 4, Switzerland

## Abstract

Semiartificial photosynthesis offers a promising route for solar chemistry, but most biohybrid systems rely on UV or blue-light excitation, are limited by inefficient interfacial charge transfer, or rely on sacrificial reagents. Here, we report a rationally designed hybrid system that integrates the broad-spectrum semiconductor BaTaO_2_N (up to ∼680 nm) with carbon nitride (CN_*x*_) and the [FeFe]-hydrogenase (H_2_ase) from *Clostridium pasteurianum* for solar-driven biomass reforming. In this architecture, BaTaO_2_N serves as a robust broad-spectrum light absorber to complement the narrow UV-blue wavelength absorption of CN_*x*_, which functions as both a photoactive component and an interfacial conduit to shuttle photogenerated electrons to [FeFe]-H_2_ase for H_2_ evolution. The optical advantage of the BaTaO_2_N|CN_*x*_|[FeFe]-H_2_ase hybrid enables markedly enhanced catalytic efficiency, achieving a H_2_ yield of 413 ± 21 μmol g^–1^ and a turnover number of 20,653 for cellulose reforming under simulated AM 1.5G irradiation at 25 °C. This work establishes an advanced design strategy for semiartificial photoreforming by combining broadband light harvesting, efficient charge transfer, and sustainable biomass valorization.

## Graphical abstract

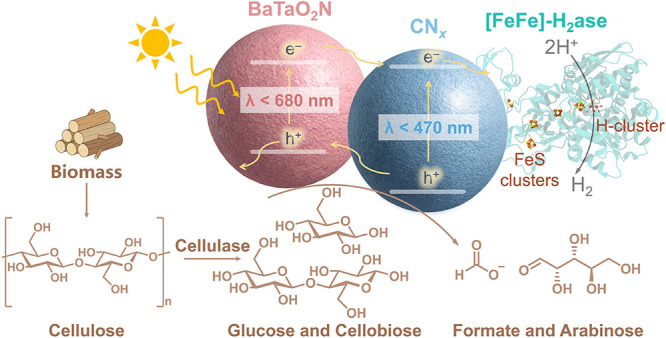

Solar reforming of waste streams offers a compelling pathway to convert solar energy into chemical fuels such as molecular hydrogen (H_2_).^1,2^ Semiartificial photosynthesis couples synthetic light absorbers with enzymes and is particularly attractive for this purpose, as it integrates robust solar harvesting with the high efficiency and selectivity of biological catalysis.^3–5^ Among enzymes, hydrogenases (H_2_ases) enable H_2_ evolution at a near-zero overpotential under mild conditions, making them ideal model catalysts for solar-driven H_2_ production.^6–8^ Despite substantial progress in H_2_ase-based biohybrid systems,^9–13^ their performance in photocatalysis remains constrained by fundamental challenges: insufficient utilization of the solar spectrum,^14–18^ inefficient interfacial electron transfer from semiconductors to enzymatic active sites, and the requirement of expensive sacrificial electron donors.^19^ While broadband photoelectrochemical platforms are available for semiartificial photosynthesis,^20,21^ overcoming these limitations simultaneously in simple photocatalytic reforming systems using biocatalysts, where solid organic waste substrates are oxidized to drive enzymatic hydrogen evolution, remains an unmet challenge.

Among broadband-responsive semiconductors, oxynitrides offer strong visible-light absorption and chemical stability.^22–24^ In particular, barium tantalum oxynitride (BaTaO_2_N) exhibits an absorption edge of ∼680 nm and high photostability,^25–28^ but semiartificial systems coupling BaTaO_2_N with enzymes have not yet been reported. This is because of directly interfacing such broadband inorganic absorbers with enzymes remains challenging due to poor interfacial compatibility and inefficient electronic coupling.^29–34^ Establishing effective charge transfer pathways without compromising broadband light harvesting thus represents a central bottleneck.

Previous interface engineering strategies have largely relied on metal oxide overlayers, such as indium tin oxide (ITO) or TiO_2_, to bridge semiconductors and enzymes (Figure 1a).^13,35^ While effective to some extent, these conventional designs treat the interfacial layer as a passive electron conduit and typically depend on wide bandgap photocatalysts and sacrificial electron donors, restricting solar utilization and broad adaptability.^35,36^

**Figure 1:**
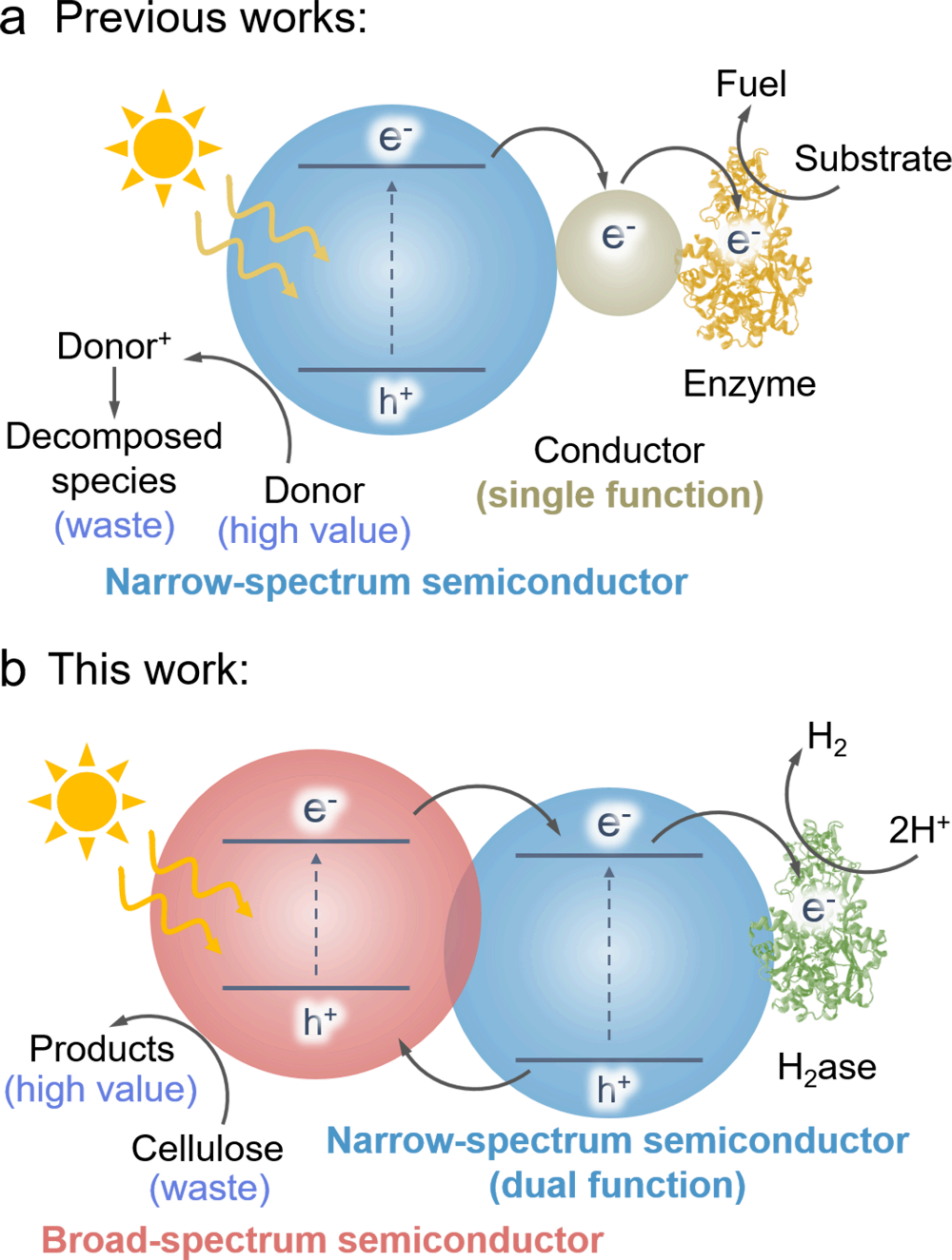
(a) Schematic illustration of commonly reported semiartificial photosynthetic systems based on wide bandgap semiconductors, sacrificial electron donors, and single-function electron conductors mediating charge transfer to enzymes. (b) Conceptual design of the present work, featuring a dual-function conduit that simultaneously facilitates interfacial electron transfer and contributes photogenerated charges, enabling solar-driven cellulose reforming coupled with enzymatic hydrogen evolution.

Here, we present a conceptually distinct strategy in which a wide bandgap semiconductor with appropriate band structure is consciously integrated with a broad-spectrum light absorber. This dual-function component not only facilitates interfacial charge transfer but also supplies additional photogenerated electrons (Figure 1b). This design enables efficient solar-driven cellulose reforming coupled with enzymatic H_2_ evolution, thereby establishing a broad-spectrum responsive and functionally integrated semiartificial photosynthetic system.

Guided by our previous findings that carbon nitride (CN_*x*_) can afford favorable electron-transfer coupling with H_2_ase and exhibits a conduction band position well-aligned for accepting electrons from BaTaO_2_N,^11,28^ we rationally selected CN_*x*_ as an interfacial conduit in a bespoke biohybrid architecture (Figure 1b and Figure 2a). The [FeFe]-H_2_ase from *Clostridium pasteurianum*, “*Cp*I”, was further chosen for its electrostatic complementarity with CN_*x*_,^37^ wherein the negatively charged CN_*x*_ surface strongly interacts with the positively charged region surrounding the distal FeS cluster, enabling direct electron transfer.^11,16^ In the resulting BaTaO_2_N|CN_*x*_|[FeFe]-H_2_ase hybrid, BaTaO_2_N extends light harvesting deep into the visible region, while CN_*x*_ operates as an active interfacial and a secondary photoresponsive absorber in the UV-blue region. This rational integration establishes a conceptually new inorganic–organic–enzyme hybrid platform that unifies broadband solar harvesting with efficient enzymatic electron utilization for biomass reforming.

**Figure 2:**
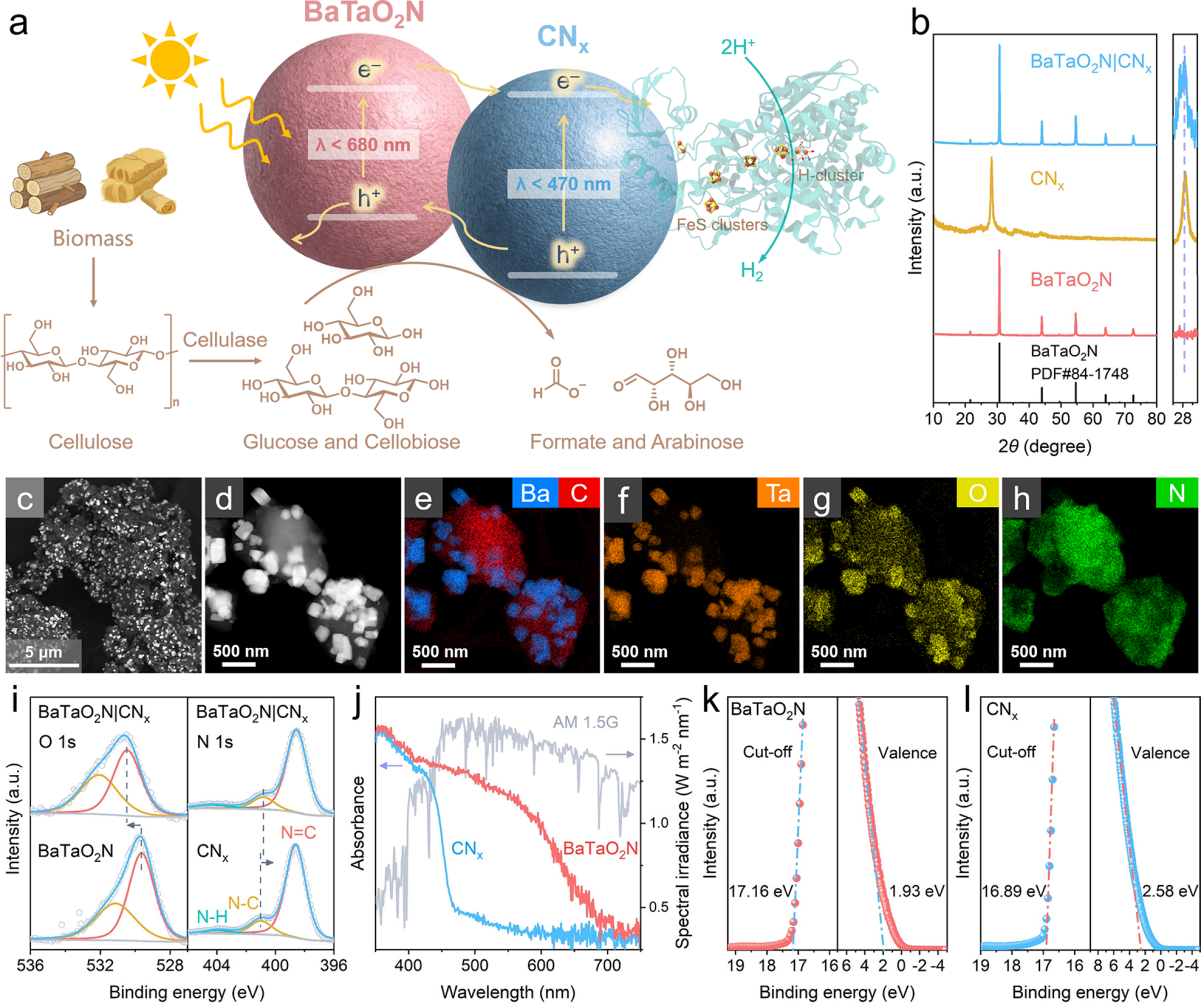
(a) Schematic illustration of the hybrid BaTaO_2_N|CN_*x*_ composite with [FeFe]-H_2_ase for cellulose photoreforming coupled to H_2_ evolution. (b) XRD pattern for BaTaO_2_N, CN_*x*_ and BaTaO_2_N|CN_*x*_. The right panel shows an enlarged view of the diffraction region around 28°. (c) SEM image in backscattered electrons mode of BaTaO_2_N|CN_*x*_. (d–h) High-Angle Annular Dark-Field (HAADF) Scanning Transmission Electron Microscopy (STEM) image in backscattered electrons mode and corresponding Energy Dispersive X-ray Spectroscopy (EDS) elemental mapping of Ba (blue), C (red), Ta (orange), O (yellow), and N (green) for BaTaO_2_N|CN_*x*_. (i) High-resolution XPS spectra of O 1s and N 1s, respectively. (j) UV–vis absorption spectra of CN_*x*_ and BaTaO_2_N, and the power spectrum of the reference sunlight (AM 1.5G). (k, l) UPS spectra of BaTaO_2_N and CN_*x*_, respectively.

BaTaO_2_N was synthesized via a flux-assisted nitridation method,^28^ and consists of well-defined cubic nanoparticles with an average size of ∼200 nm (Figures S1 and S2). CN_*x*_ was prepared from melamine and potassium thiocyanate following reported procedures,^11^ and appears as agglomerated nanosheets (Figures S3 and S4). The BaTaO_2_N|CN_*x*_ composite was obtained by mixing the two components followed by annealing, representing the first integration of BaTaO_2_N with CN_*x*_ to form a functional inorganic–organic heterostructure. X-ray diffraction (XRD) of the composite confirms the coexistence of both phases (Figure 2b), while electron microscopy and elemental mapping reveal uniform dispersion of BaTaO_2_N on CN_*x*_ (Figures 2c–h and Figures S5–S8) and intimate interfacial contact between the two semiconductors (Figures 2d–h, Figures S9 and S10).

X-ray photoelectron spectroscopy (XPS) survey spectra confirm the presence of Ba, Ta, O, N, and C in the BaTaO_2_N|CN_*x*_ composite (Figure S11). Upon hybridization, the O 1s, Ba 3d, and Ta 4f peaks of BaTaO_2_N exhibit positive binding energy shifts (Figure 2i and Figure S12), while the N 1s peak of CN_*x*_ shifts to lower binding energy with the C 1s signal remaining nearly unchanged (Figure 2i and Figure S13). These systematic shifts indicate strong interfacial electronic interactions and suggest spontaneous electron redistribution from BaTaO_2_N to CN_*x*_,^38,39^ consistent with the formation of a well-aligned heterojunction.^40,41^ UV–vis absorption and ultraviolet photoelectron spectroscopy (UPS) measurements further reveal that BaTaO_2_N exhibits broader visible-light absorption (∼680 nm) and higher conduction and valence band edge positions (−0.32 and 1.49 eV) than CN_*x*_ (−0.21 and 2.41 eV) (Figures 2j–2l, Figures S14 and S15), supporting thermodynamically favorable directional electron transfer from photoexcited BaTaO_2_N to CN_*x*_.

We next evaluated the photocatalytic activity of the BaTaO_2_N|CN_*x*_ heterostructure in enzymatic H_2_ evolution using sodium ascorbate as a sacrificial electron donor. In a typical experiment, H_2_ase (40 pmol) was interfaced with the photocatalyst (2 mg) in 1 mL of N_2_-saturated aqueous solution containing sodium ascorbate (0.1 M) and MOPS buffer (0.1 M, pH 7). The mixture was irradiated under simulated AM 1.5G light in a sealed reactor with 3.6 mL headspace at 25 °C. Control experiments show that BaTaO_2_N alone, even in the presence of H_2_ase, generates only negligible H_2_ (Figure 3a). Quartz crystal microbalance (QCM) measurements confirm that enzyme immobilization on BaTaO_2_N is less efficient than on CN_*x*_ (Figure S16),^11^ indicating that the lack of catalytic activity in the BaTaO_2_N|H_2_ase system arises not only from inefficient interfacial electron transfer between BaTaO_2_N and H_2_ases, but also from insufficient enzyme immobilization on BaTaO_2_N.

**Figure 3:**
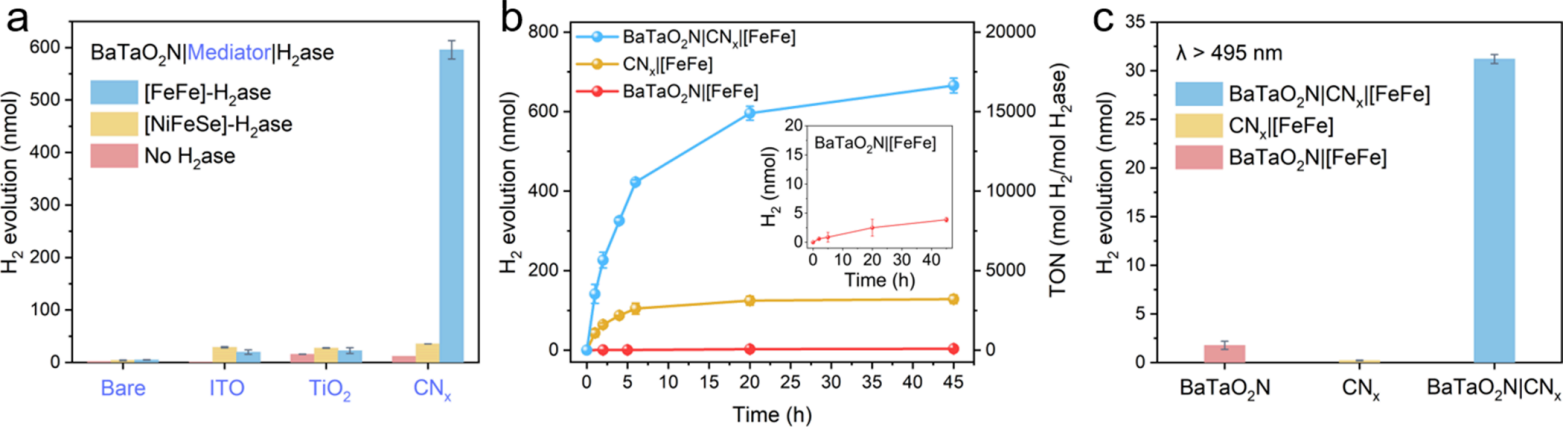
(a) Comparison of the photocatalytic performance of BaTaO_2_N using various conduits coupled with different H_2_ases after 20 h of reaction. (b) Time-dependent photocatalytic H_2_ evolution of BaTaO_2_N, CN_*x*_, and BaTaO_2_N|CN_*x*_ with [FeFe]-H_2_ase. (c) Photocatalytic performance of BaTaO_2_N, CN_*x*_, and BaTaO_2_N|CN_*x*_ under λ > 495 nm light irradiation after reaction for 20 h. Reaction conditions: 2 mg photocatalyst, 40 pmol H_2_ase, 1 mL N_2_-saturated 0.1 M sodium ascorbate and 0.1 M MOPS buffer (pH 7) aqueous solution, AM 1.5G irradiation, 650 rpm stirring, 25 °C.

In contrast, introducing CN_*x*_ as an electron conduit markedly enhances photocatalytic activity when coupled with [FeFe]-H_2_ase, delivering 298 ± 9 μmol H_2_ g^–1^ with a turnover number (TON) of 14,894 after 20 h, far exceeding those of BaTaO_2_N|[FeFe]-H_2_ase (2.5 ± 0.1 μmol H_2_ g^–1^, TON = 127) or CN_*x*_|[FeFe]-H_2_ase (62.4 ± 5.6 μmol H_2_ g^–1^, TON = 3,119) under identical conditions (Figure 3a and Figure S17). Accordingly, the BaTaO_2_N|CN_*x*_ composite exhibits substantially accelerated and sustained H_2_ evolution relative to either component (Figure 3b), evidencing the establishment of an efficient interfacial charge transfer pathway. Whereas, replacing [FeFe]-H_2_ase with [NiFeSe]-H_2_ase from *Nitratidesulfovibrio vulgaris* Hildenborough results in notably reduced activity (17.7 ± 0.01 μmol H_2_ g^–1^, TON = 883; Figure 3a), which is attributed to unfavorable electrostatic interactions between CN_*x*_ and the negatively charged distal FeS cluster of the [NiFeSe]-H_2_ase.^11,42^ Furthermore, control experiments performed in the absence of the photocatalyst, light, or sacrificial donor yield negligible H_2_ evolution (Figure S18), confirming that all components are essential for catalytic activity. Variation of electron donor concentration shows minimal impact on catalytic efficiency (Figure S19), whereas hydrogen evolution activity scales with enzyme loading and light intensity (Figures S20 and S21), indicating that the system is limited by both enzyme availability and photogenerated charge supply. In addition, the photocatalytic activity decreases over time, which can be primarily attributed to partial enzyme deactivation. Notably, replenishment of fresh [FeFe]-H_2_ase restores the hydrogen evolution activity (Figure S22), indicating that enzyme stability is an important factor influencing the overall system performance.

For comparison, ITO and TiO_2_ were also evaluated as interfacial conduits instead of CN_*x*_ between BaTaO_2_N and H_2_ase. Although both materials have been employed as electron conductors in prior semiartificial systems,^13,35^ their incorporation led to only marginal activity enhancements, irrespective of whether [NiFeSe]- or [FeFe]-H_2_ase was used (Figure 3a and Figure S23). This limited improvement likely originates from interfacial mismatches at the semiconductor–conduit or conduit–enzyme junctions, which impede efficient charge transfer.^31,43^ In contrast, the rational pairing of CN_*x*_ with [FeFe]-H_2_ase enables synergistic electronic coupling, underpinning the superior performance of the BaTaO_2_N|CN_*x*_|[FeFe]-H_2_ase system.

To probe the charge transfer mechanism, wavelength-dependent photocatalytic experiments were conducted. Under long-wavelength illumination (λ > 495 nm), where only BaTaO_2_N is photoexcited, BaTaO_2_N or CN_*x*_ on their own shows negligible H_2_ evolution, whereas the BaTaO_2_N|CN_*x*_ composite remains catalytically active (Figure 3c and Figure S24). It should be noted that the markedly reduced activity under λ > 495 nm irradiation does not reflect the intrinsic contribution of BaTaO_2_N under full-spectrum conditions, but rather arises from the limited photon flux and the absence of effective interfacial charge separation. This observation indicates that photogenerated electrons in BaTaO_2_N cannot be directly delivered to the enzyme, while coupling with CN_*x*_ enables effective electron migration from photoexcited BaTaO_2_N to CN_*x*_ and subsequent transfer to the H_2_ase (Figure S25). Such behavior is consistent with interfacial charge separation via a type-II heterojunction, while the possible contribution from a Z-scheme pathway cannot be entirely excluded and may coexist under full spectrum illumination (Figure S26).

Next, the sacrificial electron donor was replaced by oxidative valorization via solar reforming (Figure 1a), including furfuryl alcohol (FFA), 1-phenylethanol (1-PE), glycerol, and glucose as substrates. Under otherwise identical conditions, the BaTaO_2_N|CN_*x*_|[FeFe]-H_2_ase composite consistently outperformed the individual components in photocatalytic H_2_ evolution across all substrates (Figure 4a and Figure S27), demonstrating the robustness and substrate versatility of the hybrid system. Notably, using glycerol as the substrate, the BaTaO_2_N|CN_*x*_|[FeFe]-H_2_ase system achieved a TON exceeding 50,000, whereas a ∼ 65-fold enhancement relative to CN_*x*_|[FeFe]-H_2_ase was observed for glucose. These results indicate that the enhanced activity is not substrate-specific but originates from the broadened light-harvesting capability and improved interfacial charge transfer enabled by the BaTaO_2_N|CN_*x*_ heterojunction.

**Figure 4:**
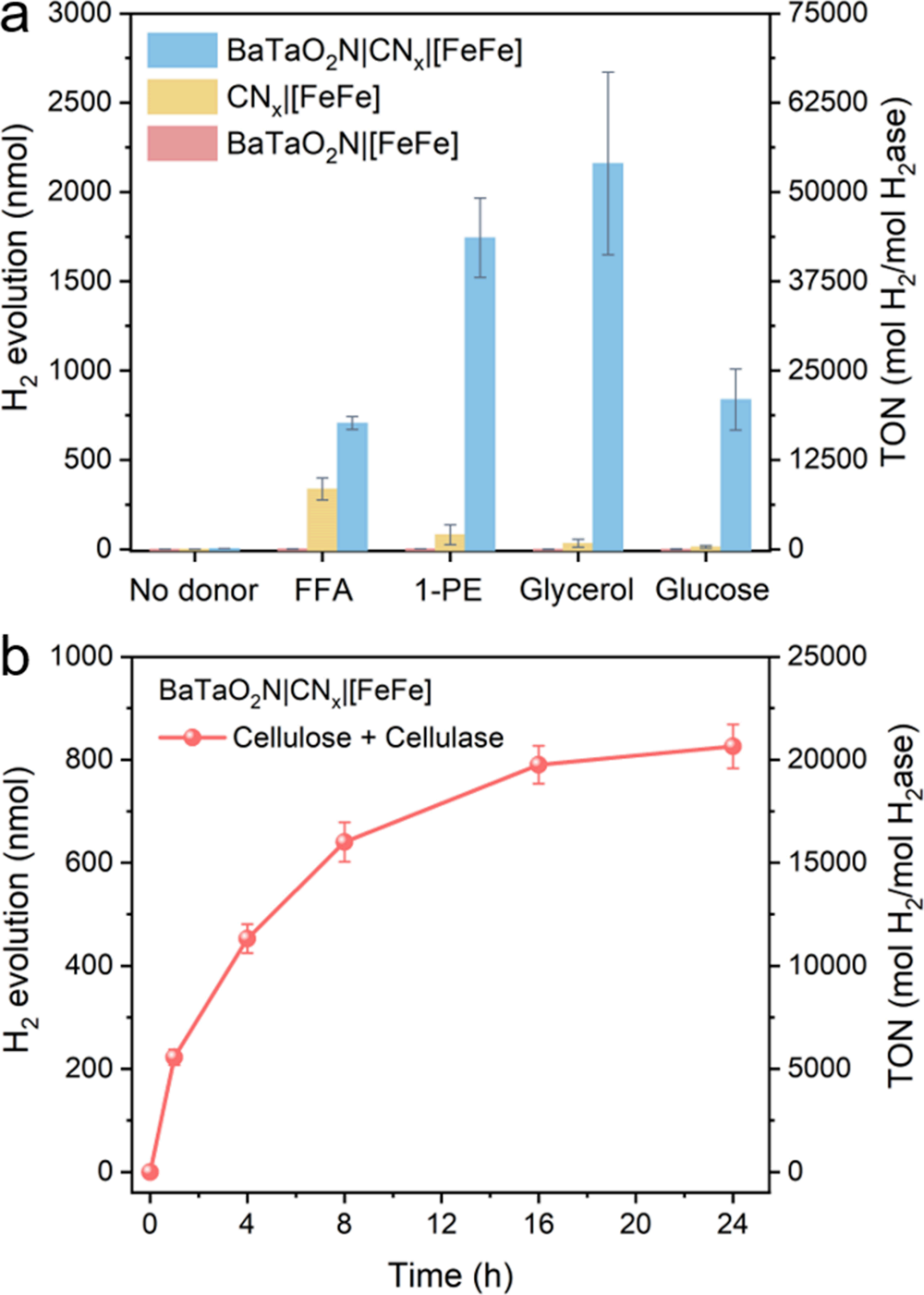
(a) Photocatalytic performance of BaTaO_2_N, CN_*x*_, and BaTaO_2_N|CN_*x*_ with [FeFe]-H_2_ase after 20 h reaction using different donor substrates. (b) Time-dependent photocatalytic H_2_ evolution of BaTaO_2_N|CN_*x*_ using pretreated cellulose. Reaction conditions: 2 mg photocatalyst, 40 pmol H_2_ase, 1 mL N_2_-saturated 0.05 M substrates (or 1 mL pretreated cellulose solution) and 0.1 M MOPS buffer (pH 7) aqueous solution, AM 1.5G irradiation, 650 rpm stirring, 25 °C.

Encouraged by the broad substrate scope, we further extended the optimized BaTaO_2_N|CN_*x*_ system to solar-driven cellulose reforming, a more sustainable and practically relevant biomass conversion process. Owing to the insolubility and recalcitrant nature of polymeric cellulose, a mild enzymatic pretreatment with cellulase was first employed to ensure effective substrate accessibility in the aqueous medium.^9^ The resulting depolymerized cellulose subsequently served as both the carbon source and electron donor, enabling coupled biomass oxidation and H_2_ evolution under solar irradiation (Figure 4b). High-performance liquid chromatography (HPLC) analysis confirmed the formation of cellobiose and glucose as the major intermediates after enzymatic hydrolysis, yielding ∼5 mM glucose (Figures S28). Upon photoreforming, oxidation products including fructose, arabinose, and formate were detected (Figures S29 and S30), indicating that cellulose conversion proceeds via sequential oxidation and C–C bond cleavage pathways (Figure S31).^9^

Most reported solar-driven biomass reforming systems rely on UV or short-wavelength visible photocatalysts such as TiO_2_,^9,44^ carbon nitride,^45^ or metal sulfides,^46^ inherently limiting solar utilization (Table S1). In contrast, the oxynitride-based architecture developed here extends light harvesting deep into the visible region (up to ∼ 680 nm), substantially expanding the usable solar spectrum. Beyond this optical advantage, the BaTaO_2_N|CN_*x*_|H_2_ase hybrid achieves 413 ± 21 μmol H_2_ g^–1^ with a TON of 20,653 for cellulose reforming under simulated solar irradiation, exceeding those of previously reported enzyme- or molecular-catalyst-based systems (Table S1). In addition to high TON values, the system exhibits competitive quantum yields (Table S1), indicating efficient photogenerated charge utilization. Together, these results highlight the power of integrating broadband oxynitride absorbers with dual-function interfacial conduits, establishing a concept for semiartificial photoreforming that unites broad-spectrum solar harvesting with efficient enzymatic biomass valorization.

In summary, we introduce a semiartificial photoreforming system that integrates a BaTaO_2_N|CN_*x*_ heterostructure with [FeFe]-H_2_ase for solar-driven H_2_ evolution coupled to biomass valorization. The biohybrid harvests visible light up to ∼680 nm, intriguingly aligning with the P680 absorption of Photosystem II and thereby extending solar utilization into the lower-energy region that matches natural photosynthesis. The well-matched heterojunction facilitates efficient interfacial charge separation and directional electron transfer to the enzymatic active site. CN_*x*_ functions as a dual-role conduit, bridging BaTaO_2_N and the enzyme while contributing additional photogenerated electrons. This rationally engineered biohybrid demonstrates broad substrate compatibility and achieves high catalytic performance. These results present an advance for semiartificial design, uniting broadband solar harvesting, efficient interfacial electron transfer, and sustainable biomass valorization.

## Supplementary Material



## Data Availability

The findings of this study are available from the University of Cambridge data repository: https://doi.org/10.17863/CAM.129517.

## Notes

Experimental details; materials synthesis and characterization; SEM images; TEM images; EDX mapping; XPS spectra; UV–vis spectra; QCM measurements; HPLC analysis; ^1^H NMR spectrum; photocatalytic activity data (Figures S1–S31, Table S1) (PDF)
