# Supplementary material for: Interfacing Broad-Spectrum Semiconductors with Hydrogenases for Semi-Artificial Solar Reforming of Cellulose
Source: J Am Chem Soc. 2026 Apr 28;148(18):18608–14. doi: 10.1021/jacs.6c03439 (PMC13185121; doi:10.1021/jacs.6c03439)
Supplement: Supplementary file 1 [file ja6c03439_si_001.pdf]

Supporting Information

Interfacing Broad-Spectrum Semiconductors with Hydrogenases for Semi-Artificial Solar Reforming of Cellulose

Ming Shi,[1,2,+] Yongpeng Liu,[1,+] Ariffin Bin Mohamad Annuar,[1] Sophie Webb,[3,4] Ross D. Milton,[3,4] Rengui Li,[2] Can Li,[2] Erwin Reisner[1,*]

[1] Yusuf Hamied Department of Chemistry, University of Cambridge, Cambridge, CB2 1EW, UK

[2] State Key Laboratory of Catalysis, Dalian National Laboratory for Clean Energy, Dalian Institute of Chemical Physics, Chinese Academy of Sciences, Dalian 116023, China

[3] Department of Inorganic and Analytical Chemistry, University of Geneva, 1211, Geneva 4, Switzerland

[4] National Centre of Competence in Research (NCCR) Catalysis, University of Geneva, 1211, Geneva 4, Switzerland

[+] Ming Shi and Yongpeng Liu contributed equally to this work.

*E-mail: reisner@ch.cam.ac.uk

Table of Contents

Experimental Section 4
Chemicals 4
Synthesis of $BaTaO_2N$ 5
Synthesis of CN_x 5
Synthesis of $BaTaO_2N|CN_x$ 5
Characterizations 5
Quartz crystal microbalance analysis 6
Pretreatment of cellulose 7
Photocatalytic reactions 7
Supporting Figures 9
Figure S1. SEM images of $BaTaO_2N$ 9
Figure S2. TEM images of $BaTaO_2N$ 10
Figure S3. SEM images of CN_x 11
Figure S4. TEM images of CN_x 12
Figure S5. SEM images of $BaTaO_2N|CN_x$ 13
Figure S6. TEM images of $BaTaO_2N|CN_x$ 14
Figure S7. SEM and corresponding EDS of $BaTaO_2N|CN_x$ 15
Figure S8. TEM and corresponding elemental mapping of $BaTaO_2N|CN_x$ 16
Figure S9. High-resolution TEM images of $BaTaO_2N|CN_x$ 17
Figure S10. High-resolution TEM and elemental mapping of $BaTaO_2N|CN_x$ 18
Figure S11. XPS survey spectra of $BaTaO_2N$, CN_x, and $BaTaO_2N|CN_x$ 19
Figure S12. High-resolution XPS spectra of Ba 3d and Ta 4f 20
Figure S13. High-resolution XPS spectra of C 1s 21
Figure S14. UV-vis absorption spectra of $BaTaO_2N$ and CN_x 22
Figure S15. Schematic band structure of $BaTaO_2N$ and CN_x 23
Figure S16. QCM analysis of the adsorption process of H_2ases on a $BaTaO_2N$ 24
Figure S17. The photocatalytic performance of CN_x with different H_2ases 25
Figure S18. Control experiments in the absence of catalysts, light, and donor 26
Figure S19. Effect of electron donor concentration on photocatalytic hydrogen evolution by the $BaTaO_2N|CN_x|$[FeFe]-H_2ase 27
Figure S20. Effect of enzyme loading on hydrogen evolution activity and TON by the $BaTaO_2N|CN_x|$[FeFe]-H_2ase 28
Figure S21. Effect of light intensity on photocatalytic hydrogen evolution by the $BaTaO_2N|CN_x|$[FeFe]-H_2ase 29

Figure S22. Enzyme re-addition experiment for the $BaTaO_2N|CN_x|$[FeFe]-H_2ase 30
Figure S23. The photocatalytic performance of ITO and TiO_2 with different H_2ases 31
Figure S24. The photocatalytic performance of $BaTaO_2N$, CN_x, and $BaTaO_2N|CN_x$ under λ > 495 nm light irradiation 32
Figure S25. Schematic diagram of photogenerated charge transfer under λ > 495 nm light excitation 33
Figure S26. Schematic illustration of photogenerated charge transfer mechanism..... 34
Figure S27. The photocatalytic performance of $BaTaO_2N$, CN_x, and $BaTaO_2N|CN_x$ under different substrates 35
Figure S28. HPLC results of cellobiose standard, glucose standard, pretreated cellulose, and calibration curve for glucose 36
Figure S29. HPLC of arabinose, fructose, and formic acid standard sample 37
Figure S30. HPLC and ^{1}H NMR spectrum of oxidation products from cellulose....... 38
Figure S31. Proposed reaction routes for cellulose conversion 39
Table S1. Comparison among photocatalytic biomass photoreforming systems 40
References 41

Experimental Section

Chemicals: All the chemicals were used as received without further purification. $BaCO_3$ (Oka, 99.99%), Ta_2O_5 (Kojundo Chemical Laboratory, 99.9%), RbCl (Wako Pure Chemical Industries, ≥95.0%), melamine (Sigma-Aldrich, 99%), potassium thiocyanate (KSCN, Sigma-Aldrich, ⩾99.0%), TiO_2 (Evonik Industries, ≥99.5%), ITO nanoparticles (<50 nm diameter, Sigma-Aldrich), 3-(N-morpholino) propanesulfonic acid (MOPS, Sigma-Aldrich, ≥99.5%), MOPS sodium salt (Sigma-Aldrich, ≥99.5%), sodium ascorbate (Thermo Scientific, 99%), furfuryl alcohol (Alfa Aesar, 98%), 1-Phenylethanol (Sigma-Aldrich, 98%), glycerol (Sigma-Aldrich, ≥99.0%), glucose (Sigma-Aldrich, ≥99.5%), cellobiose (Acros Organics, 98%), fructose (Fischer Scientific, 99%), and arabinose (Thermo Scientific, 99%) were used for all the experiments.

The [FeFe]-H_2ase from *Clostridium pasteurianum*, "*CpI*", was heterologously expressed in *Escherichia coli* BL21(DE3) Δ*iscR* cells with a C-terminal Strep-tag, with in vivo maturation enabled by co-expression of HydEFGX maturases from *Shewanella oneidensis*. Cells were lysed under strictly anaerobic conditions, and the enzyme was purified from the soluble fraction using StrepTactin-XT affinity chromatography, followed by concentration (~5 mg mL^{-1}) via ultrafiltration in a stirred cell concentrator (Millipore). Protein concentration was determined by the Biuret assay (using BSA as the standard), and purity was confirmed by SDS-PAGE. The catalytic activity was evaluated under anaerobic conditions in MOPS buffer (pH 7) using methyl viologen (10 mM) as the electron mediator and sodium dithionite (100 mM) as the electron donor. Hydrogen production was quantified by gas chromatography after incubation at 37 °C. The enzyme exhibited high proton reduction activity, with specific activities of 1031–2790 μmol H_2 min^{-1} mg^{-1}, confirming its excellent catalytic performance.[1, 2]

The recombinant [NiFeSe]-H_2ase was produced in *Nitratidesulfovibrio vulgaris* Hildenborough and purified under anaerobic conditions. Cells were grown in modified Postgate medium, harvested at late exponential phase, and disrupted under an N_2 atmosphere. The soluble fraction was isolated by ultracentrifugation and purified by sequential Q-Sepharose anion-exchange and Strep-Tactin affinity chromatography,

followed by concentration using ultrafiltration. Protein concentration was determined by a bicinchoninic acid (BCA) assay. The catalytic activity of [NiFeSe]-H_2ase was evaluated under anaerobic conditions via both hydrogen evolution and hydrogen oxidation assays. Hydrogen production was quantified by gas chromatography, while hydrogen oxidation activity was measured spectrophotometrically using methyl viologen as the electron acceptor. The purified enzyme exhibited high catalytic activity, with an H_2 production rate of 8,280 ± 382 s^{-1} (5,640 ± 260 U mg^{-1}), confirming its excellent catalytic performance.[3]

Synthesis of $BaTaO_2N$: $BaTaO_2N$ crystals were prepared via a flux-assisted one-pot nitridation method.[4, 5] Typically, $BaCO_3$ and Ta_2O_5 (Ba/Ta molar ratio = 1.10) were combined with RbCl flux at a solute concentration of 10 mol%. The resulting mixture was thoroughly ground in an agate mortar for 30 min, placed in an alumina crucible, and subjected to nitridation at 950 °C for 8 h under a continuous NH_3 (200 mL min^{-1}). After the reaction, the products were rinsed with hot water to eliminate residual flux and subsequently dried overnight in vacuum at 40 °C.

Synthesis of CN_x: 5 g of melamine was placed in a lidded ceramic crucible and calcined in air at 550 °C for 3 h with a heating rate of 1 °C min^{-1}.[6] The resulting solid was subsequently combined with potassium thiocyanate at a mass ratio of 1:2. This mixture was transferred into a ceramic boat and subjected to a two-step thermal treatment under an Ar atmosphere of first at 400 °C for 1 h and then at 500 °C for 30 min, with a heating ramp of 30 °C min^{-1}.[7] After cooling to room temperature, the obtained material was sequentially washed twice with water and once with a water/ethanol mixture (1:1), followed by drying at ambient conditions overnight, yielding the final CN_x powder.

Synthesis of composite photocatalyst: The $BaTaO_2N$ powder was thoroughly mixed with either CN_x, TiO_2 or ITO powder in an agate mortar at a 1:1 mass ratio, followed by heat treatment at 300 °C for 1 h with a heating rate of 10 °C min^{-1}, yielding the composite catalyst.

Characterizations: The powder X-ray diffraction (PXRD) were recorded on Malvern Panalytical Empyrean Series 2 diffractometer using Cu-Kα radiation. The scan rate of

5° min^{-1} was applied to record the XRD patterns in the range of 5–80°. The morphology and elemental analysis of the samples were collected using scanning electron microscopy (SEM) TESCAN CLARA 2 FEG-SEM and Oxford Instruments X-maxN 80 EDS system and transmission electron microscopy (TEM) Thermo Fisher Scientific Talos F200X G2 TEM equipped with Ceta camera for TEM imaging, bright field (BF) and high angle annular dark field (HAADF) scanning transmission electron microscopy (STEM) detectors and a Super-X energy dispersive X-Ray spectroscopy (EDS) detector system. X-ray photoelectron spectroscopy (XPS) characterizations were carried out on a Thermo Scientific Escalab 250Xi spectrometer with monochromatized aluminum Kα. Ultraviolet photoelectron spectroscopy (UPS) measurements were tested on Thermofisher Escalab 250Xi spectrometer with monochromatized He I photon energy (21.22 eV) excitation, and C1s (284.8 eV) was used to calibrate the peak positions of various elements. UV–visible spectra were carried out on the Cary 60 UV–vis spectrometer over the spectral range of 200–800 nm. A Waters Breeze high-performance liquid chromatography (HPLC) system equipped with a refractive index detector (RID-2414) and a diode array UV-vis detector operated at 210 and 254 nm was employed to analyze the oxidation conversion and product yield on a proton column maintained at 40 °C under isocratic conditions, employing 5 mM H_2SO_4 as the mobile phase with a flow rate of 0.5 mL min^{-1}. The proton nuclear magnetic resonance (^{1}H NMR) spectra were collected with a Bruker 400 MHz NMR spectrometer at 25 °C.

Quartz crystal microbalance (QCM): QCM experiments were conducted on a Biolin Q-Sense Explorer module and a custom-designed QCM flow cell within an anaerobic glovebox (MBraun, N_2 atmosphere, <0.1 ppm of O_2). A gold-coated quartz chip with a surface area of 0.79 cm^2 and a surface roughness <1 nm RMS was utilized. The chip was functionalized by drop-casting an ultrasonicated suspension (0.1 mL) of $BaTaO_2N$ (0.5 mg mL^{-1}) in isopropyl alcohol, forming a thin layer on the surface. Once the baseline reached a steady state, 80 pmol of enzyme (either [FeFe]-H_2ase or [NiFeSe]-H_2ase) was introduced into the 2 mL MOPS buffer solution (pH 7). The adsorption of the enzyme onto the surface was quantified by monitoring changes in the resonance frequency of the piezoelectric quartz chip. To determine the corresponding mass change,

the change in frequency (Δf) was analyzed using the Sauerbrey equation:

$$\Delta f = -\frac{2f_0^2}{A\sqrt{p_q \mu_q}}\Delta m$$

where f_0 is the resonance frequency (5 MHz) of the quartz oscillator, A is the piezoelectrically active crystal area, Δm is the change in mass, p_q is the density of quartz, and μ_q is the shear modulus of quartz. Assuming 25% of the adsorbed mass consisted of water molecules bound to the enzymes, Δm can be converted into quantity of enzymes.

Pretreatment of cellulose: Cellulose was pretreated with cellulase according to a previously reported procedure.[8] Briefly, 2.5 g of cellulose was suspended in 50 mL of 0.1 M $NaHCO_3$ solution (pH 6.5, adjusted with HCl) and maintained at 37 °C. Subsequently, 0.5 g of cellulose in 50 mL of the same $NaHCO_3$ buffer (pH 6.5) was added to the suspension. The mixture was incubated at 37 °C for 24 h under continuous stirring, after which the unreacted cellulose was removed by filtration through a 0.2 μm syringe filter. The resulting filtrate was stored at −4 °C prior to use. The pretreated cellulose solution contained about 5 mM glucose as determined by HPLC.

Photocatalytic reactions: Photocatalyst samples (2 mg) were dispersed in 1 mL of an aqueous solution containing 0.1 M MOPS buffer (pH 7) and the corresponding substrates, followed by loaded into a sealed reactor with a headspace volume of 3.6 mL and ultrasonication for 5 min prior to the addition of 40 pmol H_2ase. All photocatalytic reactions were assembled and sealed inside an anaerobic glovebox (MBraun, N_2 atmosphere, < 0.1 ppm O_2). During reaction, the reactors were illuminated under simulated sunlight (AM 1.5G, 100 mW cm^{-2}) generated by a Newport xenon arc lamp housing (66921) and stirred at 650 rpm, and the temperature of reactor was maintained at 25 °C by a cooling water system. The generated hydrogen was quantified using a gas chromatograph (Shimadzu Tracera GC-2010 Plus, TCD, Ar carrier). The hydrogen evolution activities reported in μmol H_2 g^{-1} are normalized based on the total mass of the semiconductor photocatalyst (2 mg) used in each experiment.

The turnover number (TON) is calculated based on the total amount of enzyme added,

defined as moles of H_2 produced per mole of enzyme.[9-11]

$$\mathrm{TON} = \frac{n_{product}(mol)}{n_{product}(mol)} = \frac{n_{H_2}(mol)}{n_{H_2ase}(mol)}$$

For the quantum yield (QY) calculation, the incident photon flux was quantified using $K_3[Fe(C_2O_4)_3]$ as a chemical actinometer, in accordance with established procedures reported in the literature.[12-14] The calculation employed the known characteristics of the Fe^{2+} complex, including a molar absorption coefficient of 11,100 M^{-1} cm^{-1} and a quantum yield of Fe^{2+} generation of 1.21.[12-14] The incident photon number was quantified as $(1.9 \pm 0.2) \times 10^{19}$ photons per hour, based on which the overall QY for proton reduction to H_2 is defined as:

$$\mathrm{QY}(\%) = \frac{2 \times n(H_2)}{Incident\ photons} \times 100$$

Supporting Figures

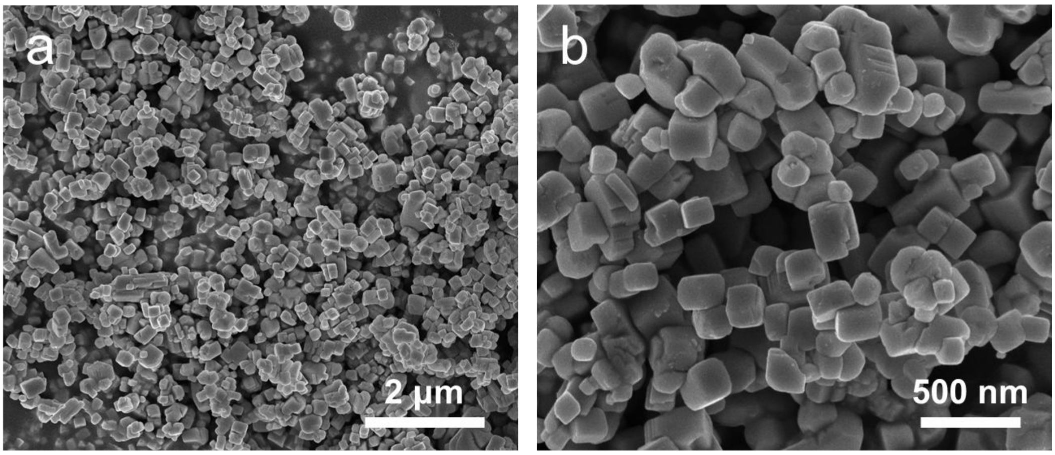

Figure S1. SEM images of $BaTaO_2N$.

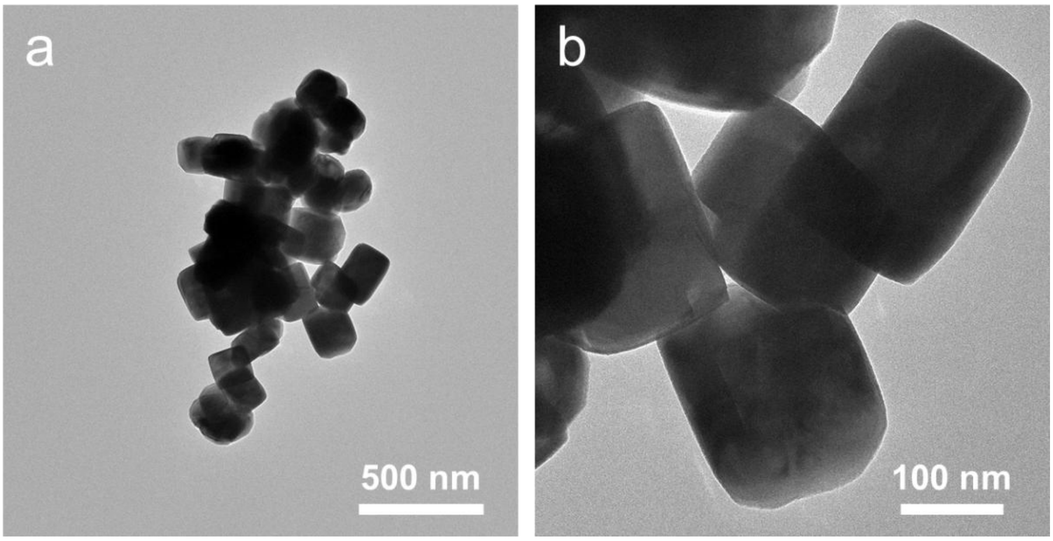

Figure S2. TEM images of $BaTaO_2N$.

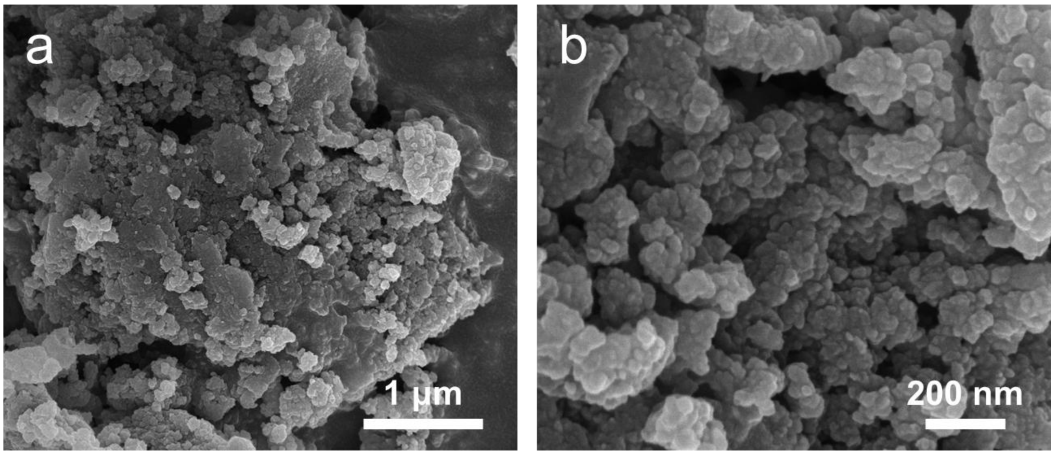

Figure S3. SEM images of CN_x.

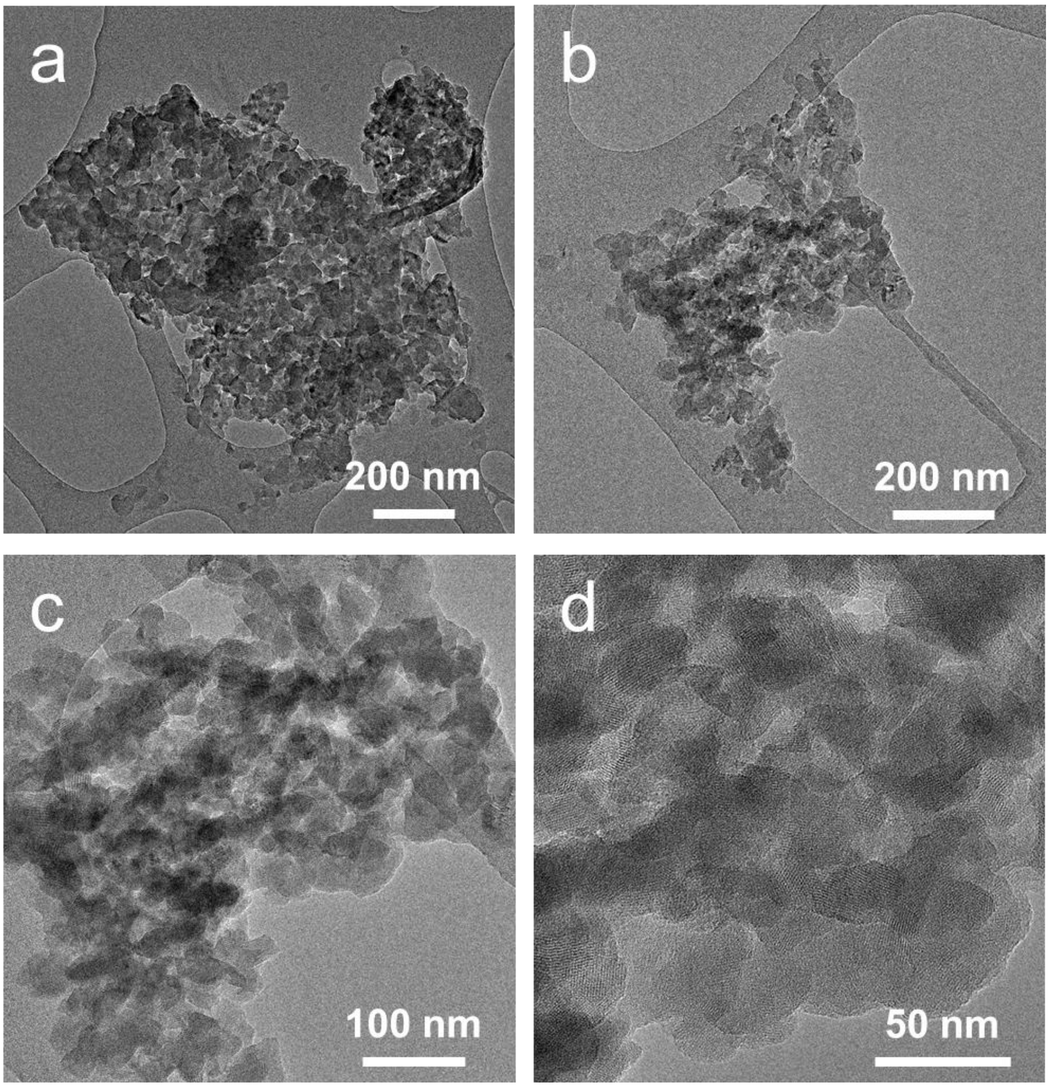

Figure S4. TEM images of CN_x.

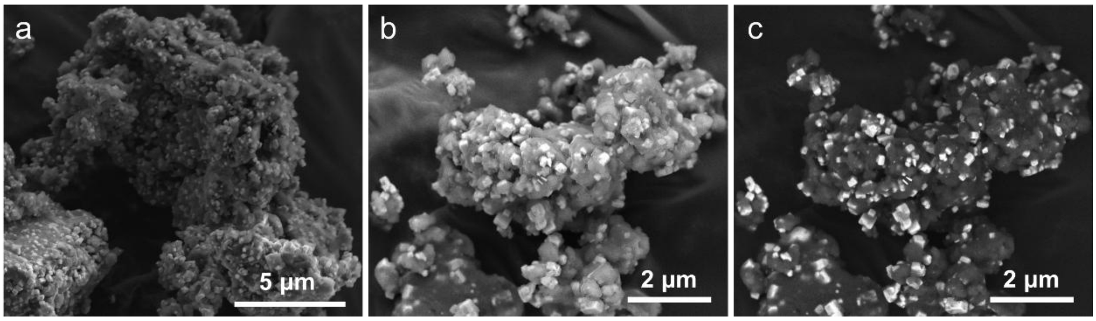

Figure S5. SEM images of $BaTaO_2N|CN_x$. The (a) and (b) are the secondary electrons (SE) images, and (c) is the backscattered electrons (BSE) image.

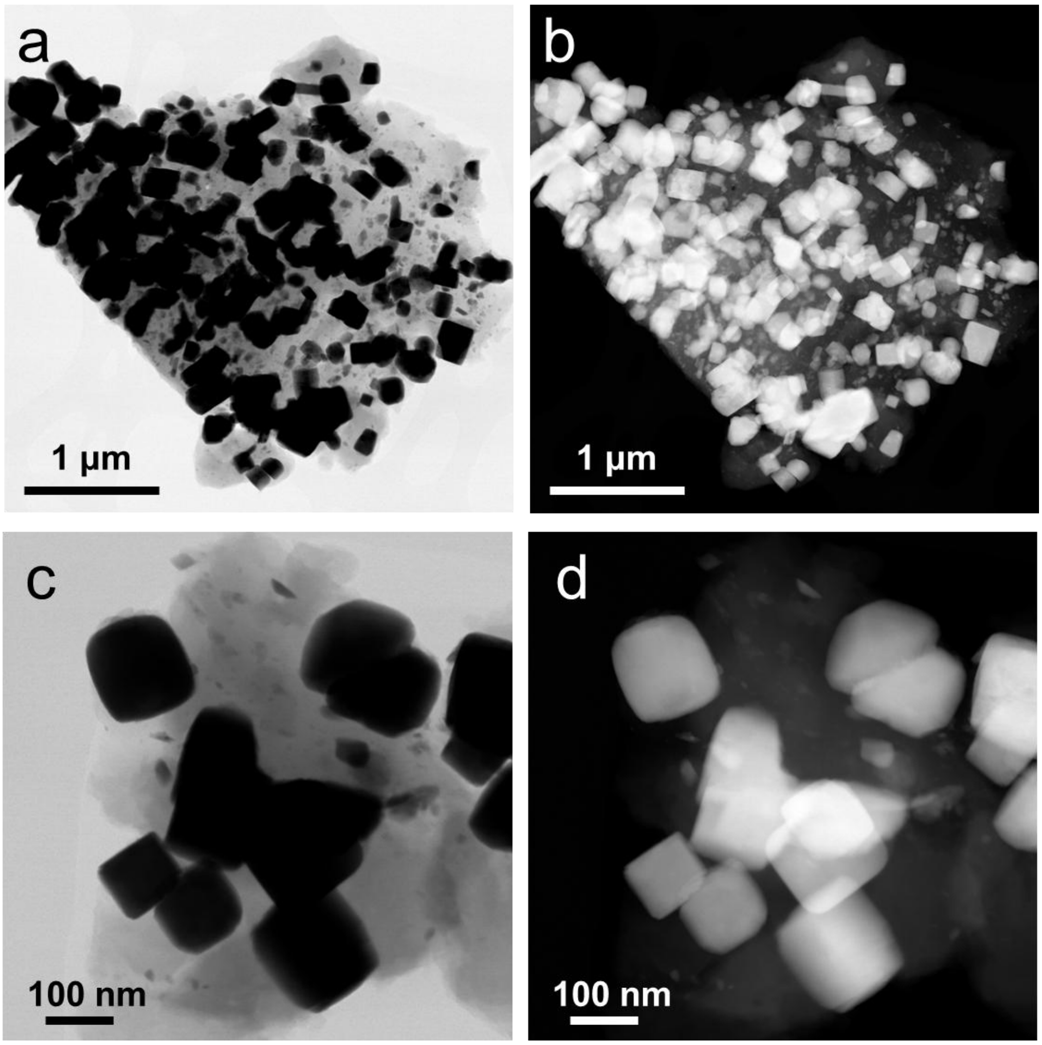

Figure S6. STEM images of $BaTaO_2N|CN_x$. (a) and (c) are the BF-STEM images, and (b) and (d) are the HAADF-STEM images.

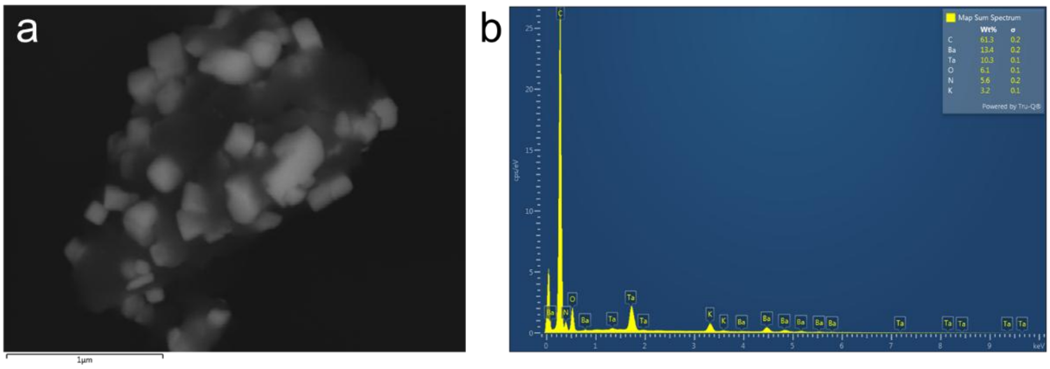

Figure S7. (a) BSE-SEM image of $BaTaO_2N|CN_x$, and (b) the corresponding energy dispersive X-ray spectroscopy (EDS) result.

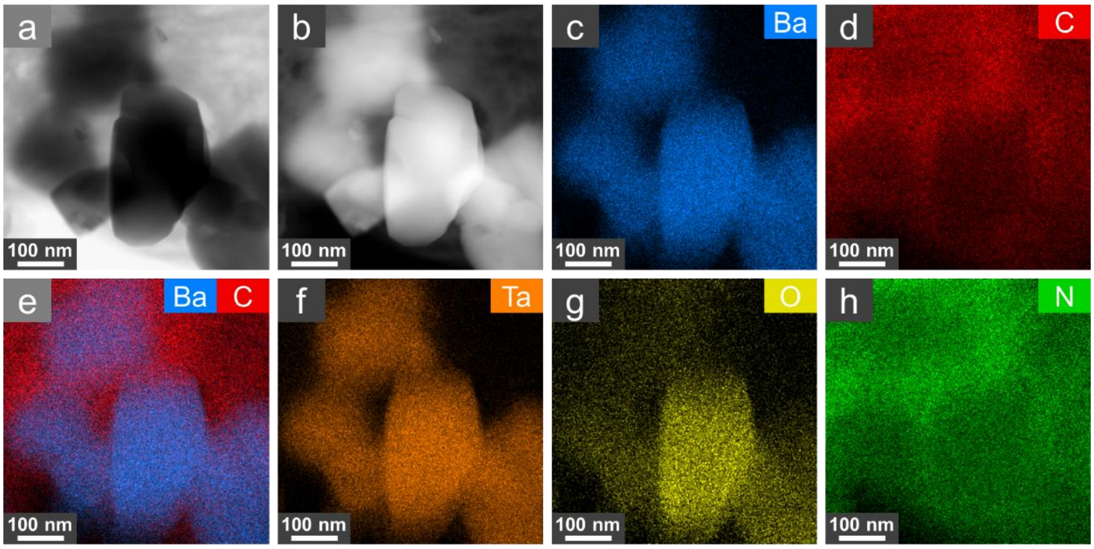

Figure S8. (a, b) BF and HAADF-STEM images, respectively, and (c–h) corresponding EDS elemental mapping of Ba (blue), C (red), Ta (orange), O (yellow), and N (green) for $BaTaO_2N|CN_x$.

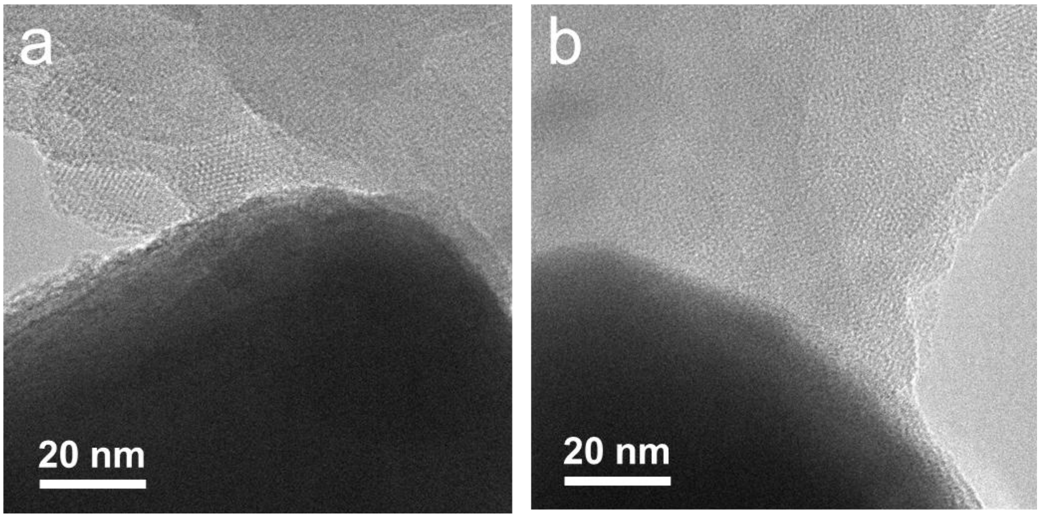

Figure S9. High-resolution TEM images of $BaTaO_2N|CN_x$.

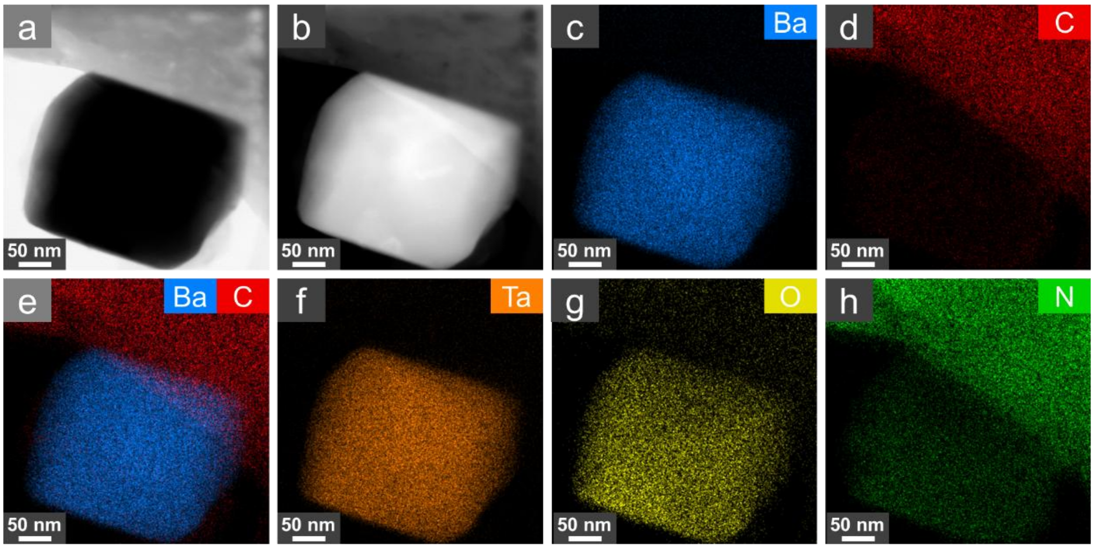

Figure S10. (a, b) High magnification BF and HAADF-STEM images, respectively, and (c–h) corresponding EDS elemental mapping of Ba (blue), C (red), Ta (orange), O (yellow), and N (green) for $BaTaO_2N|CN_x$.

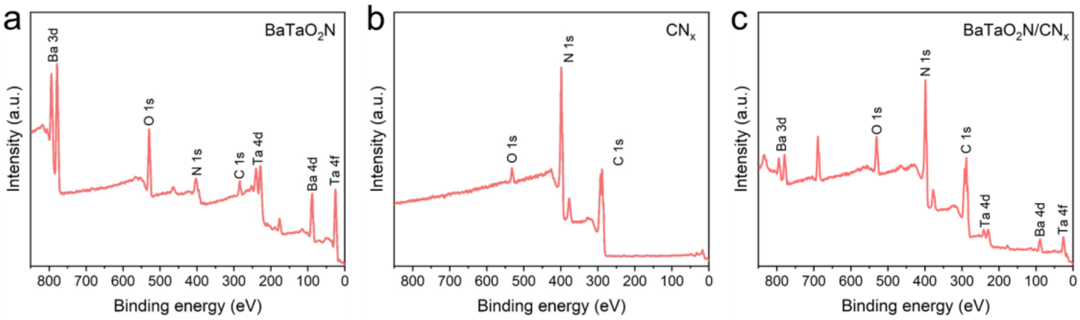

Figure S11. XPS survey spectra of (a) $BaTaO_2N$, (b) CN_x, and (c) $BaTaO_2N|CN_x$.

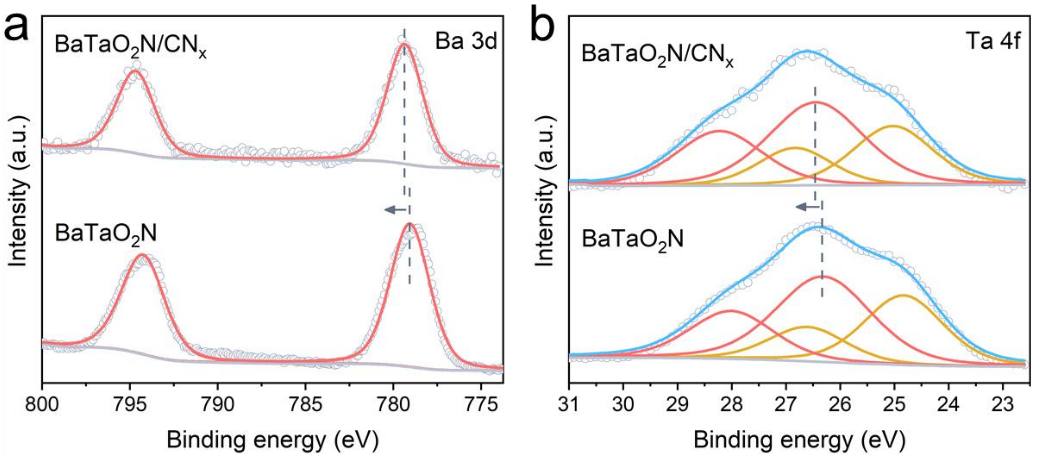

Figure S12. High-resolution XPS spectra of (a) Ba 3d and (b) Ta 4f for $BaTaO_2N$ and $BaTaO_2N|CN_x$, respectively.

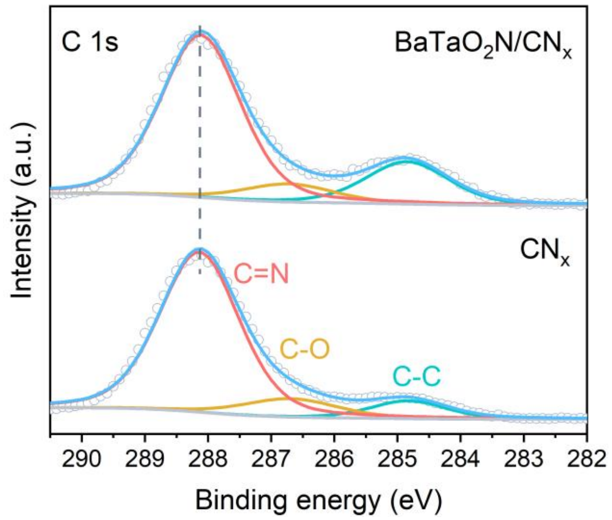

Figure S13. High-resolution XPS spectra of C 1s for CN_x and $BaTaO_2N|CN_x$, respectively.

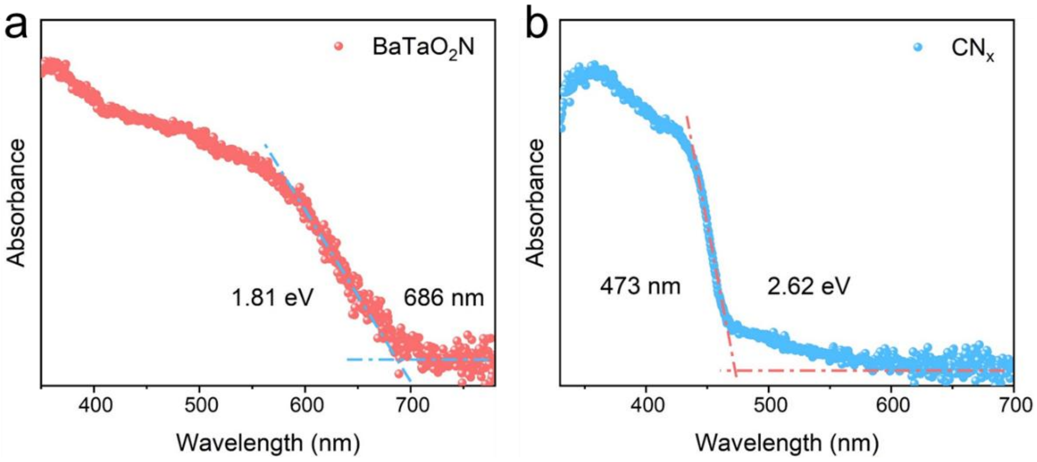

Figure S14. UV–vis absorption spectra of (a) $BaTaO_2N$ and (b) CN_x, respectively.

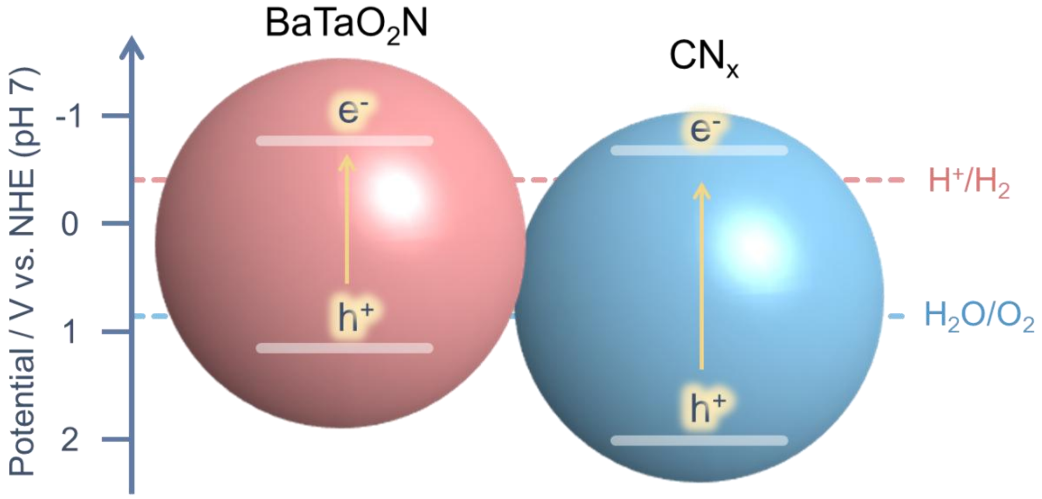

Figure S15. Schematic band structure of $BaTaO_2N$ and CN_x.

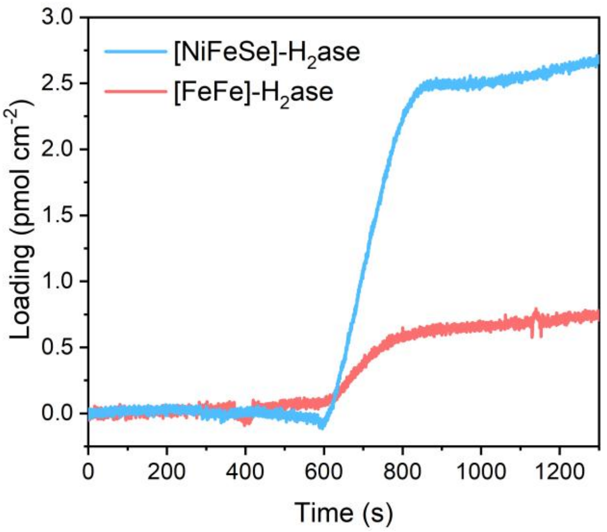

Figure S16. QCM analysis of the adsorption process of H_2ases on a $BaTaO_2N$-coated quartz chip. Loading conditions: 0.141 mL min^{-1} flow rate, an anaerobic aqueous solution (2 mL, pH 7) containing MOPS (0.1 M), and 80 pmol of enzymes (either [FeFe]-H_2ase or [NiFeSe]-H_2ase), 25 °C.

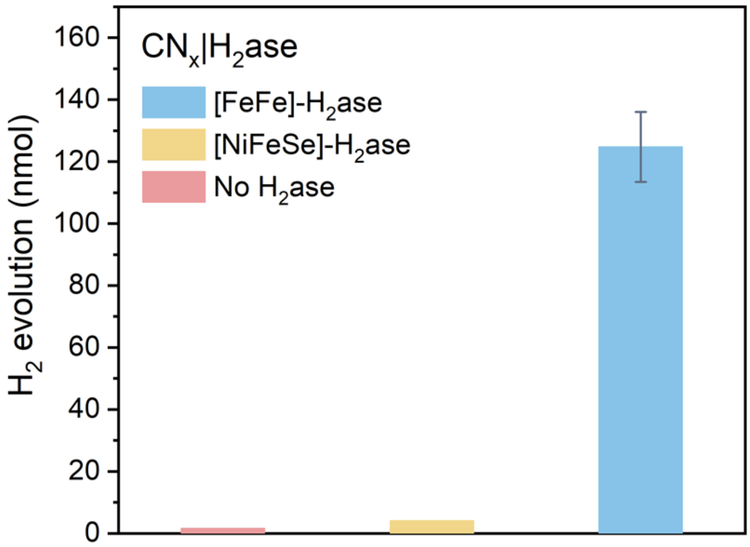

Figure S17. The photocatalytic performance of CN_x with different hydrogenase. Reaction conditions: 2 mg photocatalyst, 40 pmol H_2ase, 1 mL N_2-saturated aqueous solution containing 0.1 M sodium ascorbate and 0.1 M MOPS buffer (pH 7), AM 1.5G irradiation, 650 rpm stirring, 25 °C, 20 h.

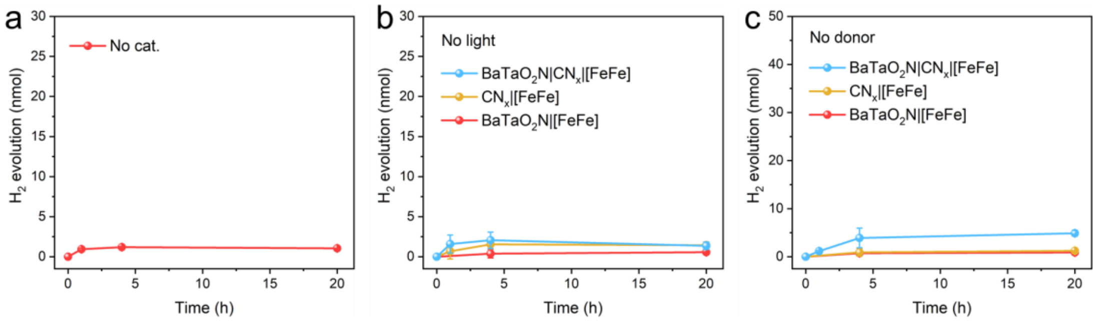

Figure S18. Exclusion control experiments in the absence of (a) catalyst, (b) light, and (c) donor. Reaction conditions: 2 mg photocatalyst, 40 pmol [FeFe]-H_2ase, 1 mL N_2-saturated aqueous solution containing 0.1 M sodium ascorbate and 0.1 M MOPS buffer (pH 7), AM 1.5G irradiation, 650 rpm stirring, 25 °C, 20 h.

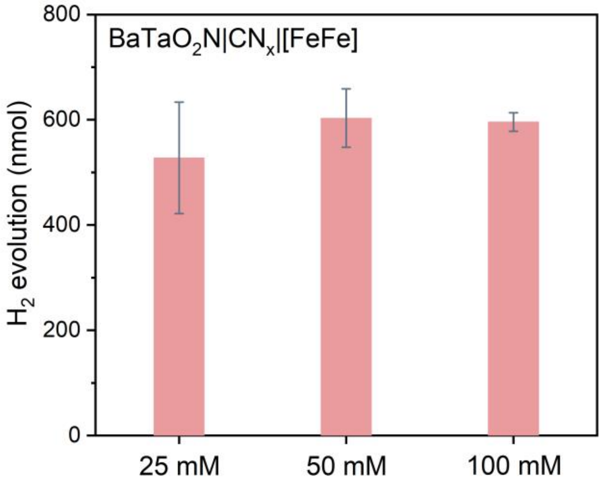

Figure S19. Effect of electron donor concentration on photocatalytic hydrogen evolution by the $BaTaO_2N|CN_x|$[FeFe]-H_2ase system. Reaction conditions: 2 mg photocatalyst, 40 pmol H_2ase, 1 mL N_2-saturated 25 mM (50 mM or 100 mM) sodium ascorbate and 0.1 M MOPS buffer (pH 7) aqueous solution, AM 1.5G irradiation, 650 rpm stirring, 25 °C, after reaction for 20 h.

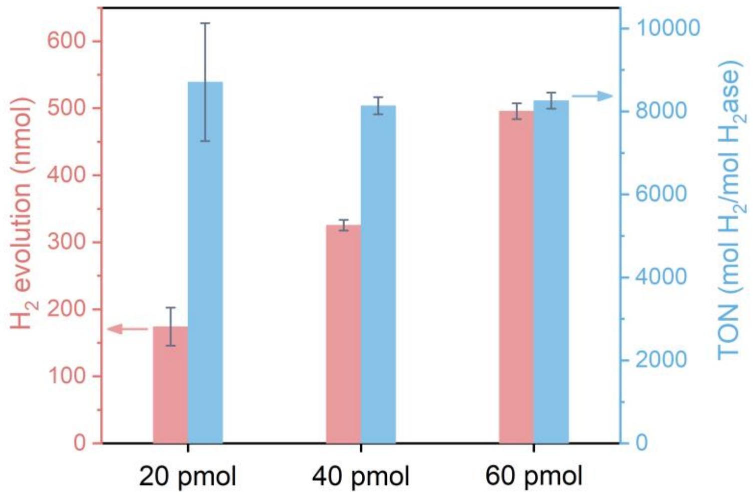

Figure S20. Effect of enzyme loading on hydrogen evolution activity and turnover number (TON) by the $BaTaO_2N|CN_x|$[FeFe]-H_2ase system. Reaction conditions: 2 mg photocatalyst, 20 pmol (40 pmol or 60 pmol) H_2ase, 1 mL N_2-saturated 0.1 M sodium ascorbate and 0.1 M MOPS buffer (pH 7) aqueous solution, AM 1.5G irradiation, 650 rpm stirring, 25 °C, after reaction for 4 h.

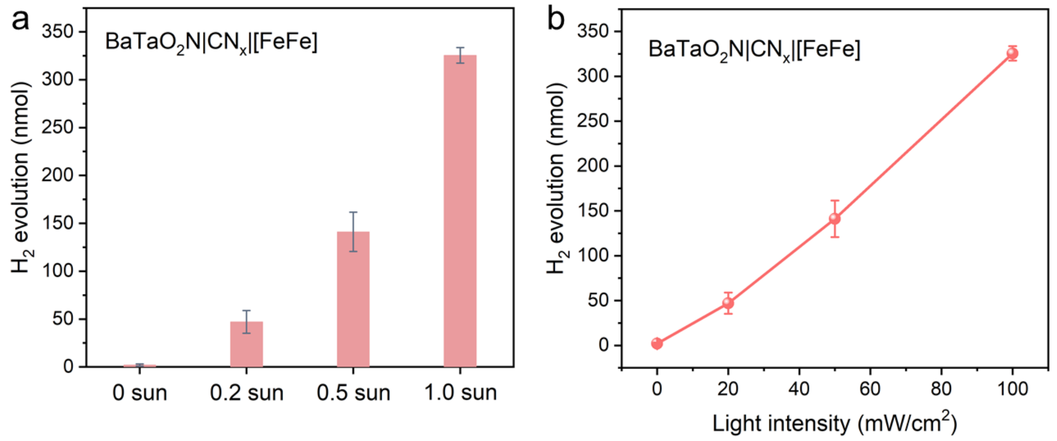

Figure S21. (a) The photocatalytic hydrogen evolution activity on different light intensity and (b) dependence of hydrogen evolution activity on light intensity by the $BaTaO_2N|CN_x|$[FeFe]-H_2ase system. Reaction conditions: 2 mg photocatalyst, 40 pmol H_2ase, 1 mL N_2-saturated 0.1 M sodium ascorbate and 0.1 M MOPS buffer (pH 7) aqueous solution, different intensity of AM 1.5G irradiation, 650 rpm stirring, 25 °C, after reaction for 4 h.

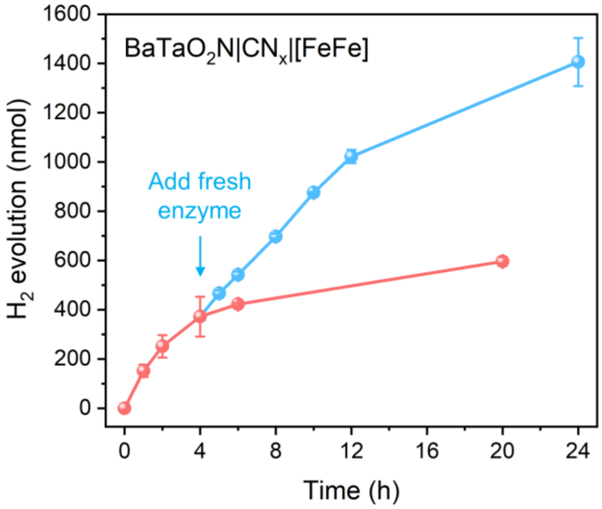

Figure S22. Enzyme re-addition experiment for the $BaTaO_2N|CN_x|$[FeFe]-H_2ase system. The red line indicates the amount of hydrogen produced without adding fresh enzyme. Reaction conditions: 2 mg photocatalyst, 40 pmol H_2ase, 1 mL N_2-saturated 0.1 M sodium ascorbate and 0.1 M MOPS buffer (pH 7) aqueous solution, AM 1.5G irradiation, 650 rpm stirring, 25 °C.

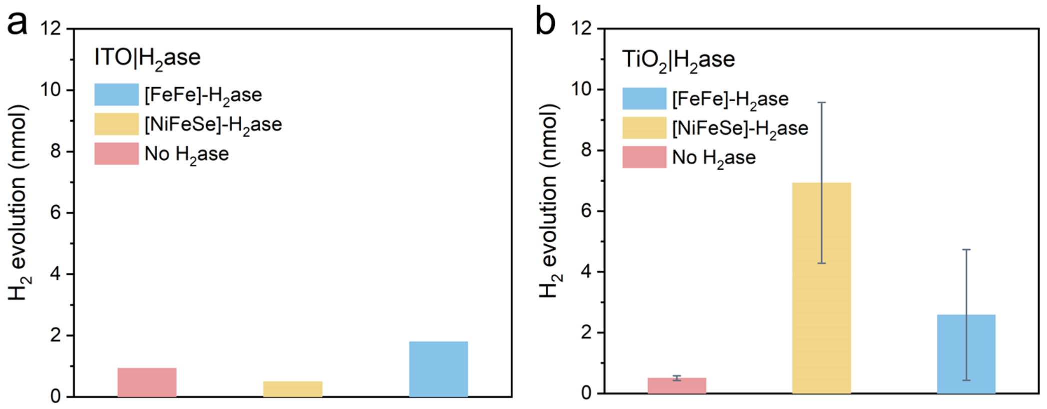

Figure S23. The photocatalytic performance of (a) ITO and (b) TiO_2 with different hydrogenase. Reaction conditions: 2 mg photocatalyst, 40 pmol H_2ase, 1 mL N_2-saturated aqueous solution containing 0.1 M sodium ascorbate and 0.1 M MOPS buffer (pH 7), AM 1.5G irradiation, 650 rpm stirring, 25 °C, 20 h.

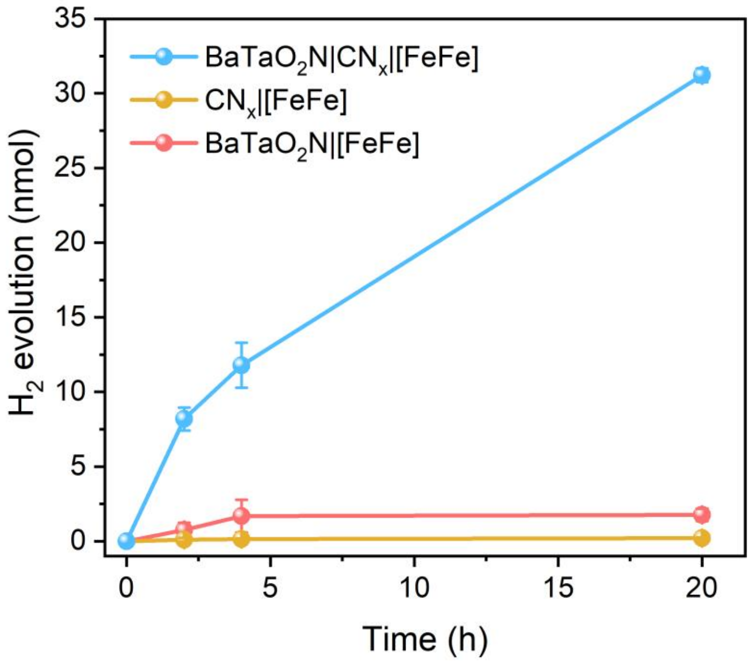

Figure S24. The photocatalytic performance of $BaTaO_2N$, CN_x, and $BaTaO_2N|CN_x$ with [FeFe]-H_2ase under λ > 495 nm light irradiation. Reaction conditions: 2 mg photocatalyst, 40 pmol H_2ase, 1 mL N_2-saturated aqueous solution containing 0.1 M sodium ascorbate and 0.1 M MOPS buffer (pH 7), AM 1.5G irradiation, 650 rpm stirring, 25 °C.

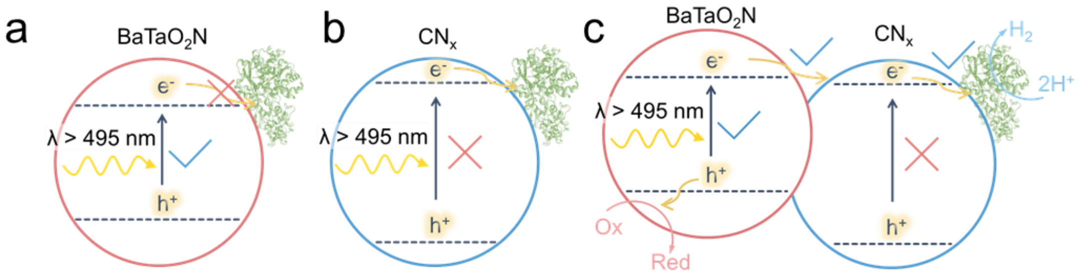

Figure S25. (a–c) Schematic diagram of photogenerated charge transfer of $BaTaO_2N$, CN_x, and $BaTaO_2N|CN_x$ under λ > 495 nm light excitation.

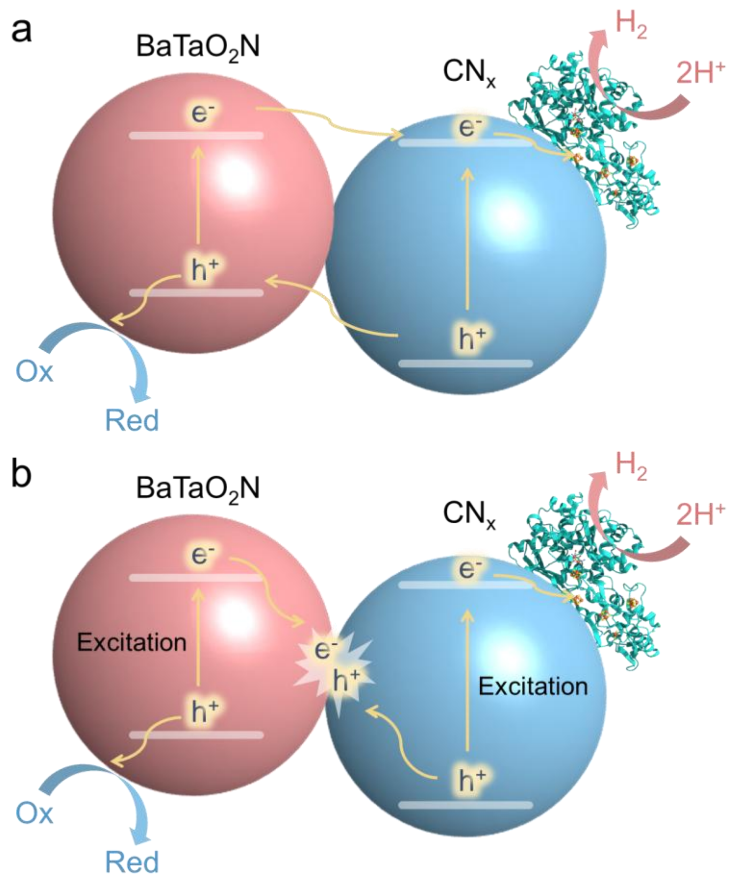

Figure S26. Schematic illustrations of (a) the proposed photogenerated charge transfer mechanism in a Type-II heterojunction and (b) an alternative Z-scheme charge transfer pathway between $BaTaO_2N$ and CN_x.

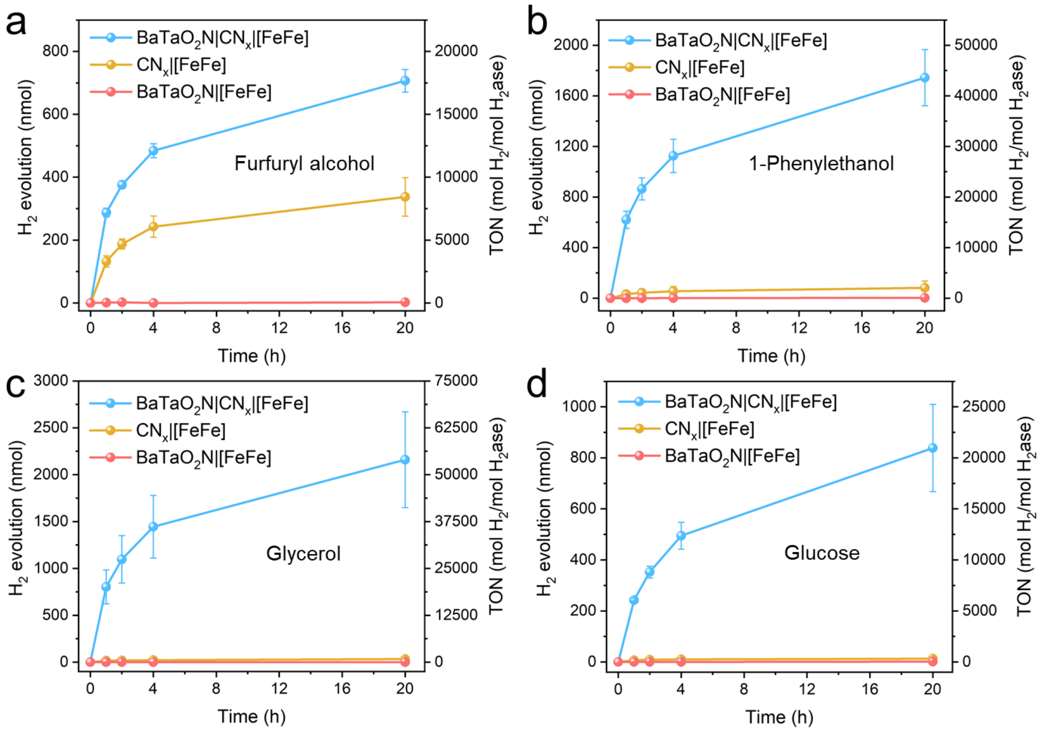

Figure S27. The photocatalytic performance of $BaTaO_2N$, CN_x, and $BaTaO_2N|CN_x$ with [FeFe]-H_2ase under different electron donors of (a) furfuryl alcohol, (b) 1-phenylethanol, (c) glycerol, and (d) glucose. Reaction conditions: 2 mg photocatalyst, 40 pmol H_2ase, 1 mL N_2-saturated aqueous solution containing 0.05 M electron donors and 0.1 M MOPS buffer (pH 7), AM 1.5G irradiation, 650 rpm stirring, 25 °C.

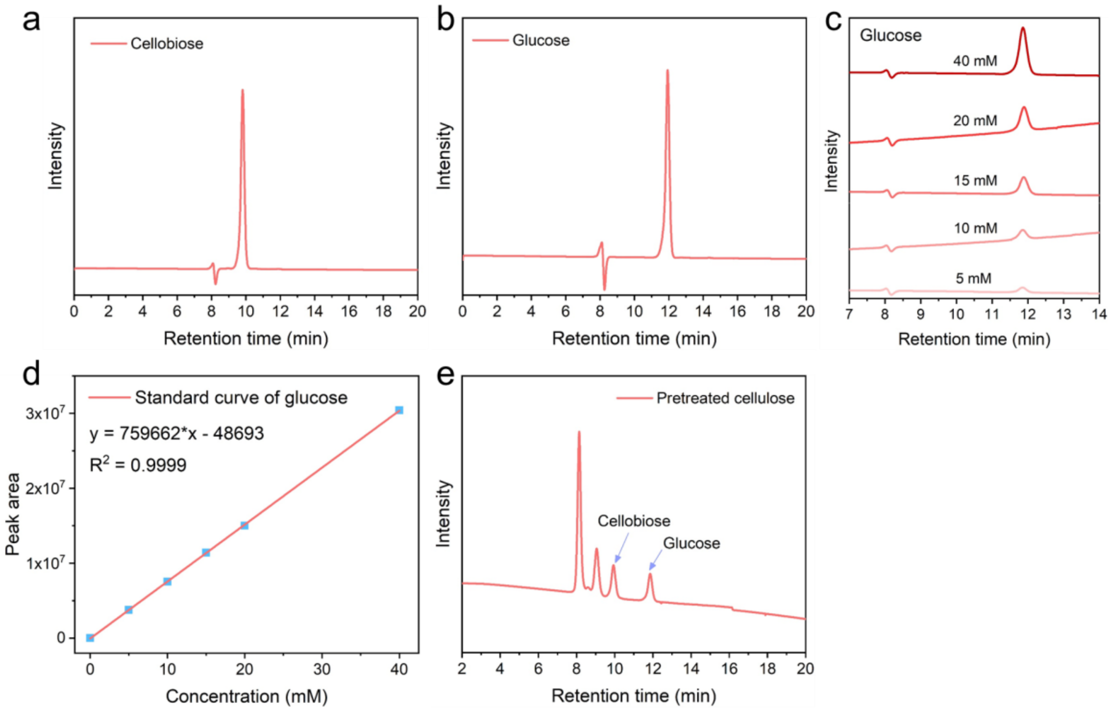

Figure S28. High-performance liquid chromatography (HPLC) analysis results: (a) cellobiose standard, (b) glucose standard, (c) glucose samples at different concentrations, and (d) corresponding calibration curve. (e) HPLC chromatogram of pretreated cellulose.

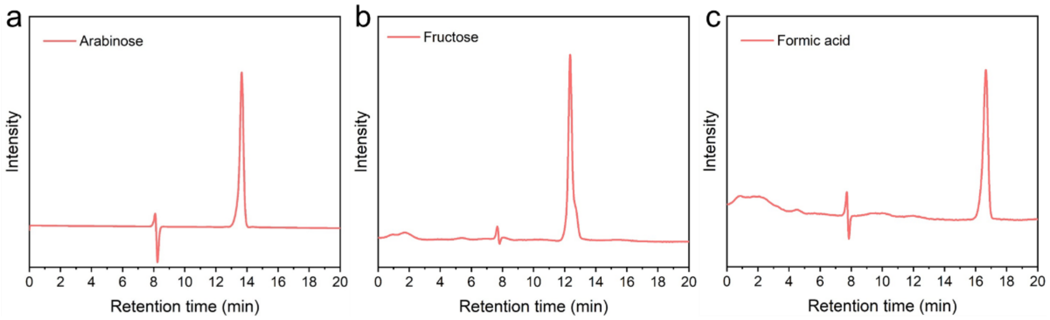

Figure S29. The HPLC of (a) arabinose, (b) fructose, and (c) formic acid standard sample.

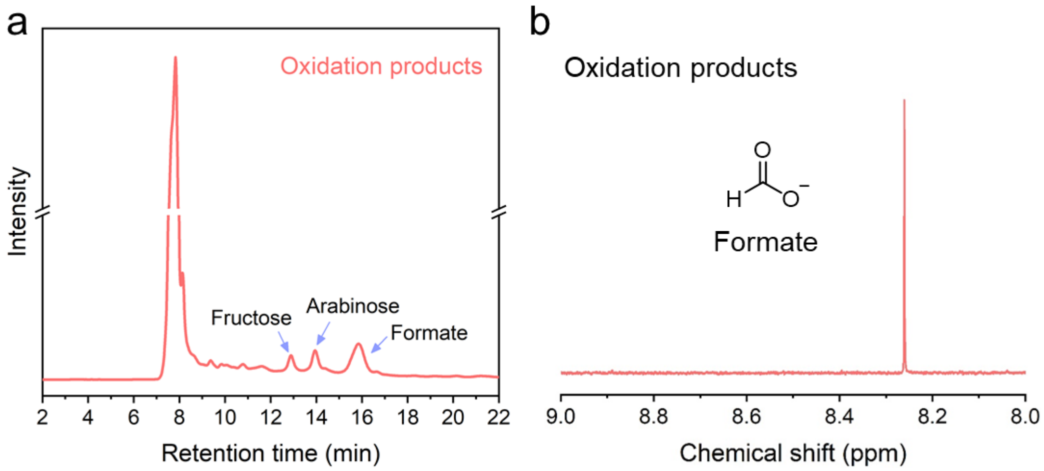

Figure S30. (a) HPLC of oxidation products from cellulose. (b) ^{1}H NMR spectrum (400 MHz, $CDCl_3$ as the internal standard) of formate from cellulose oxidation.

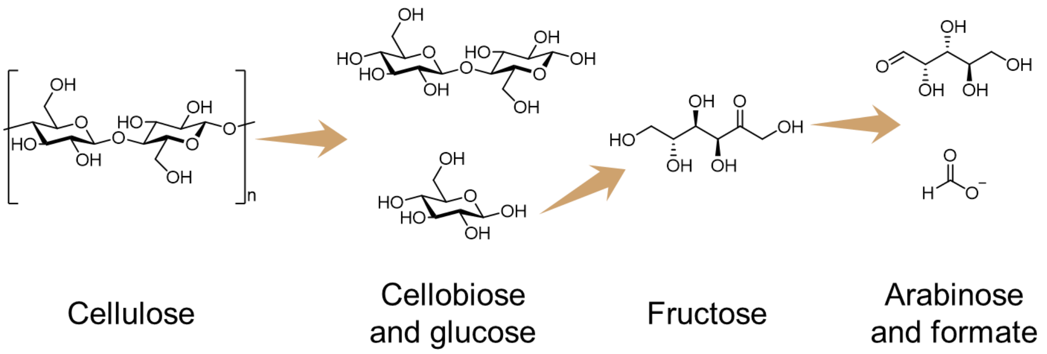

Figure S31. The proposed reaction routes for cellulose conversion.

Table S1. Comparison among state-of-the-art photocatalytic biomass photoreforming systems.

Photocatalytic system	Light absorption range	Substrate	TON	QY	Ref.
$BaTaO_2N\|CN_x\|H_2ase$	< 680 nm	Pretreated cellulose	20653	1.4%	This work
$TiO_2\|H_2ase$	< 400 nm	Glucose	87	-	8
$TiO_2\|NiP$	< 400 nm	Glucose	0.3	-	8
$ZnIn_2S_4\|NiP$	< 560 nm	2-phenoxy-1-phenylethanol	120	1.3%	15
$CN_x\|NiP$	< 450 nm	Glucose	642	-	16
$TiO_2\|CotpyP$	< 400 nm	Glucose	75	1.2%	17
$TiO_2\|CotpyP$	< 400 nm	Pretreated cellulose	38	-	17
Carbon dots|NiP	visible-light	Cellulose	101	-	18
Carbon dots|NiP	visible-light	Glucose	121	-	18

References

(1) Webb, S.; Veliju, A.; Maroni, P.; Apfel, U. P.; Happe, T.; Milton, R. D. Mesoporous Electrodes Enhance the Electrocatalytic Performance of [FeFe]-Hydrogenase. *Angew. Chem. Int. Ed. Engl.* **2025,** *64*, e202416658.

(2) Liu, Y.; Webb, S.; Moreno-Garcia, P.; Kulkarni, A.; Maroni, P.; Broekmann, P.; Milton, R. D. Facile Functionalization of Carbon Electrodes for Efficient Electroenzymatic Hydrogen Production. *JACS Au* **2023,** *3*, 124-130.

(3) Marques, M. C.; Tapia, C.; Gutierrez-Sanz, O.; Ramos, A. R.; Keller, K. L.; Wall, J. D.; De Lacey, A. L.; Matias, P. M.; Pereira, I. A. C. The direct role of selenocysteine in [NiFeSe] hydrogenase maturation and catalysis. *Nat. Chem. Biol.* **2017,** *13*, 544-550.

(4) Wang, Z.; Luo, Y.; Hisatomi, T.; Vequizo, J. J. M.; Suzuki, S.; Chen, S.; Nakabayashi, M.; Lin, L.; Pan, Z.; Kariya, N.; Yamakata, A.; Shibata, N.; Takata, T.; Teshima, K.; Domen, K. Sequential cocatalyst decoration on $BaTaO_2N$ towards highly-active Z-scheme water splitting. *Nat. Commun.* **2021,** *12*, 1005.

(5) Luo, Y.; Wang, Z.; Suzuki, S.; Yubuta, K.; Kariya, N.; Hisatomi, T.; Domen, K.; Teshima, K. Fabrication of Single-Crystalline $BaTaO_2N$ from Chloride Fluxes for Photocatalytic H_2 Evolution under Visible Light. *Cryst. Growth Des.* **2019,** *20*, 255-261.

(6) Liu, J.; Liu, Y.; Liu, N.; Han, Y.; Zhang, X.; Huang, H.; Lifshitz, Y.; Lee, S. T.; Zhong, J.; Kang, Z. Water splitting. Metal-free efficient photocatalyst for stable visible water splitting via a two-electron pathway. *Science* **2015,** *347*, 970-974.

(7) Lau, V. W.; Moudrakovski, I.; Botari, T.; Weinberger, S.; Mesch, M. B.; Duppel, V.; Senker, J.; Blum, V.; Lotsch, B. V. Rational design of carbon nitride photocatalysts by identification of cyanamide defects as catalytically relevant sites. *Nat. Commun.* **2016,** *7*, 12165.

(8) Lam, E.; Miller, M.; Linley, S.; Manuel, R. R.; Pereira, I. A. C.; Reisner, E. Comproportionation of CO_2 and Cellulose to Formate Using a Floating Semiconductor-Enzyme Photoreforming Catalyst. *Angew. Chem. Int. Ed.* **2023,** *62*, e202215894.

(9) Wang, F.; Wen, M.; Feng, K.; Liang, W. J.; Li, X. B.; Chen, B.; Tung, C. H.; Wu, L. Z. Amphiphilic polymeric micelles as microreactors: improving the photocatalytic hydrogen production of the [FeFe]-hydrogenase mimic in water. *Chem. Commun.* **2016,** *52*, 457-460.

(10) Troppmann, S.; Brandes, E.; Motschmann, H.; Li, F.; Wang, M.; Sun, L.; König, B. Enhanced Photocatalytic Hydrogen Production by Adsorption of an [FeFe]-Hydrogenase Subunit Mimic on Self-Assembled Membranes. *Eur. J. Inorg. Chem.* **2016,** *2016*, 554-560.

(11) Liu, Y.; Pulignani, C.; Webb, S.; Cobb, S. J.; Rodriguez-Jimenez, S.; Kim, D.; Milton, R. D.; Reisner, E. Electrostatic [FeFe]-hydrogenase-carbon nitride assemblies for efficient solar hydrogen production. *Chem. Sci.* **2024,** *15*, 6088-6094.

(12) Hatchard, C. G.; Parker, C. A. A new sensitive chemical actinometer-II. Potassium ferrioxalate as a standard chemical actinometer. *Proc. R. Soc. Lond. A.* **1956,** *235*, 518-536.

(13) Yu, J.; Huang, L.; Tang, Q.; Yu, S.-B.; Qi, Q.-Y.; Zhang, J.; Ma, D.; Lei, Y.; Su, J.; Song, Y.; Eloi, J.-C.; Harniman, R. L.; Borucu, U.; Zhang, L.; Zhu, M.; Tian, F.; Du,

L.; Phillips, D. L.; Manners, I.; Ye, R.; Tian, J. Artificial spherical chromatophore nanomicelles for selective CO_2 reduction in water. *Nat. Catal.* **2023**, *6*, 464-475.

(14) Rao, H.; Schmidt, L. C.; Bonin, J.; Robert, M. Visible-light-driven methane formation from CO_2 with a molecular iron catalyst. *Nature* **2017**, *548*, 74-77.

(15) Chen, L.; Liu, Y.; Mitra, S.; Kim, D.; Huang, Z.; Vahey, D. M.; Bin Mohamad Annuar, A.; Reisner, E. Solar Lignin Reforming with Tunable Selectivity Using a Hybrid Photocatalyst in Aqueous Solution. *J. Am. Chem. Soc.* **2025,** *147*, 43509-43516.

(16) Kasap, H.; Achilleos, D. S.; Huang, A.; Reisner, E. Photoreforming of Lignocellulose into H_2 Using Nanoengineered Carbon Nitride under Benign Conditions. *J. Am. Chem. Soc.* **2018,** *140*, 11604-11607.

(17) Lam, E.; Reisner, E. A TiO_2-Co(terpyridine)$_2$ Photocatalyst for the Selective Oxidation of Cellulose to Formate Coupled to the Reduction of CO_2 to Syngas. *Angew. Chem. Int. Ed.* **2021,** *60*, 23306-23312.

(18) Achilleos, D. S.; Yang, W.; Kasap, H.; Savateev, A.; Markushyna, Y.; Durrant, J. R.; Reisner, E. Solar Reforming of Biomass with Homogeneous Carbon Dots. *Angew. Chem. Int. Ed.* **2020,** *59*, 18184-18188.

End of Supporting Information
